# Acute kidney injury in patients with cirrhosis: Acute Disease Quality Initiative (ADQI) and International Club of Ascites (ICA) joint multidisciplinary consensus meeting

**DOI:** 10.1016/j.jhep.2024.03.031

**Published:** 2024-03-26

**Authors:** Mitra K. Nadim, John A. Kellum, Lui Forni, Claire Francoz, Sumeet K. Asrani, Marlies Ostermann, Andrew S. Allegretti, Javier A. Neyra, Jody C. Olson, Salvatore Piano, Lisa B. VanWagner, Elizabeth C. Verna, Ayse Akcan-Arikan, Paolo Angeli, Justin M. Belcher, Scott W. Biggins, Akash Deep, Guadalupe Garcia-Tsao, Yuri S. Genyk, Pere Gines, Patrick S. Kamath, Sandra L. Kane-Gill, Manish Kaushik, Nuttha Lumlertgul, Etienne Macedo, Rakhi Maiwall, Sebastian Marciano, Raimund H. Pichler, Claudio Ronco, Puneeta Tandon, Juan-Carlos Q. Velez, Ravindra L. Mehta, François Durand

**Affiliations:** 1 Division of Nephrology and Hypertension, Keck School of Medicine, University of Southern California, Los Angeles, USA;; 2 Center for Critical Care Nephrology, University of Pittsburgh, Pittsburgh, PA, USA;; 3 School of Medicine, University of Surrey and Critical Care Unit, Royal Surrey Hospital Guildford UK;; 4 Hepatology & Liver Intensive Care, Hospital Beaujon, Clichy, Paris, France;; 5 Baylor University Medical Center, Dallas, Texas, USA;; 6 King’s College London, Guy’s & St Thomas’ Hospital, Department of Critical Care, London, UK;; 7 Division of Nephrology, Department of Medicine, Massachusetts General Hospital, Boston, MA, USA;; 8 Division of Nephrology, Department of Medicine, University of Alabama at Birmingham, Birmingham, Alabama, USA;; 9 Division of Gastroenterology and Hepatology, Mayo Clinic, Rochester, Minnesota, USA;; 10 Unit of Internal Medicine and Hepatology, Department of Medicine – DIMED, University and Hospital of Padova, Padova, Italy;; 11 Division of Digestive and Liver Diseases, University of Texas Southwestern Medical Center, Dallas, Texas, USA;; 12 Division of Digestive and Liver Diseases, Columbia University, New York, NY, USA;; 13 Department of Pediatrics, Divisions of Critical Care Medicine and Nephrology, Baylor College of Medicine, Houston, TX, USA;; 14 Unit of Internal Medicine and Hepatology, University and Teaching Hospital of Padua, Italy;; 15 Section of Nephrology, Department of Internal Medicine, Yale University School of Medicine, New Haven, CT, USA;; 16 VA Connecticut Healthcare System, West Haven, CT, USA;; 17 Division of Gastroenterology, Department of Medicine, University of Pittsburgh, Pittsburgh, PA, USA;; 18 Pediatric Intensive Care Unit, King’s College Hospital, London, UK;; 19 Digestive Diseases Section, Yale University School of Medicine, New Haven, CT, USA;; 20 Division of Abdominal Organ Transplantation and Hepatobiliary Surgery, Keck School of Medicine, University of Southern California, Los Angeles, CA, USA;; 21 Division of Abdominal Organ Transplantation at Children’s Hospital of Los Angeles, Los Angeles, CA, USA;; 22 Liver Unit, Hospital Clínic de Barcelona, University of Barcelona, Institut d’Investigacions Biomèdiques August Pi-Sunyer and Ciber de Enfermedades Hepàticas y Digestivas, Barcelona, Catalonia, Spain;; 23 Division of Gastroenterology and Hepatology Mayo Clinic College of Medicine and Science, Rochester, MN, USA;; 24 Department of Pharmacy and Therapeutics, School of Pharmacy, University of Pittsburgh, Pittsburgh, PA, USA;; 25 Department of Renal Medicine, Singapore General Hospital, Singapore;; 26 Excellence Centre in Critical Care Nephrology and Division of Nephrology, Faculty of Medicine, King Chulalongkorn Memorial Hospital, Bangkok, Thailand;; 27 Division of Nephrology, Department of Medicine, University of California San Diego, CA, USA;; 28 Department of Hepatology, Institute of Liver and Biliary Sciences, New Delhi, India;; 29 Liver Unit, Hospital Italiano de Buenos Aires, Buenos Aires, Argentina;; 30 Division of Nephrology, Department of Medicine, University of Washington, Seattle, WA, USA;; 31 International Renal Research Institute of Vicenza, Department of Nephrology, Dialysis and Transplantation, San Bortolo Hospital, Vicenza-Italy;; 32 Division of Gastroenterology (Liver Unit), University of Alberta, Edmonton, Alberta, Canada;; 33 Department of Nephrology, Ochsner Health, New Orleans, LA, USA;; 34 Ochsner Clinical School, The University of Queensland, Brisbane, QLD, Australia;; 35 Division of Nephrology-Hypertension, Department of Medicine, University of California San Diego, La Jolla, CA, USA;; 36 University Paris Cité, Paris, France

**Keywords:** hepatorenal syndrome, acute kidney injury, liver transplantation, acute disease quality initiative, international club of ascites, cirrhosis, biomarker, renal replacement therapy, ascites, albumin, terlipressin

## Abstract

Patients with cirrhosis are prone to developing acute kidney injury (AKI), a complication associated with a markedly increased in-hospital morbidity and mortality, along with a risk of progression to chronic kidney disease. Whereas patients with cirrhosis are at increased risk of developing any phenotype of AKI, hepatorenal syndrome (HRS), a specific form of AKI (HRS-AKI) in patients with advanced cirrhosis and ascites, carries an especially high mortality risk. Early recognition of HRS-AKI is crucial since administration of splanchnic vasoconstrictors may reverse the AKI and serve as a bridge to liver transplantation, the only curative option. In 2023, a joint meeting of the International Club of Ascites (ICA) and the Acute Disease Quality Initiative (ADQI) was convened to develop new diagnostic criteria for HRS-AKI, to provide graded recommendations for the work-up, management and post-discharge follow-up of patients with cirrhosis and AKI, and to highlight priorities for further research.

## Introduction

Acute kidney injury (AKI) occurs in up to 60% of hospitalized patients with cirrhosis and is associated with increased morbidity and mortality.^1–9^ In 2012, the Acute Disease Quality Initiative (ADQI) VIII and the International Club of Ascites (ICA) proposed diagnostic criteria for AKI^10^ which were further revised in 2015 by the ICA.^11^ Over the last decade, there have been significant advances in the field.^12^ In 2023, a joint meeting of ADQI (ADQI XXIX) and the ICA was reconvened to refine the diagnostic criteria for AKI and hepatorenal syndrome (HRS), review their epidemiology and pathophysiology, explore the role of biomarkers in the diagnosis and prognostication of AKI, examine current and novel therapies for the prevention and treatment of AKI, and create a potential paradigm for the post-discharge care of patients who experience AKI or acute kidney disease (AKD), especially as they progress to chronic kidney disease (CKD). The goals of the meeting were to provide recommendations for clinical practice and identify knowledge gaps to inform a research framework for this clinically important area.

## Methods

The ADQI-ICA consensus conference chairs (MKN, FD and RLM) convened a diverse international panel of clinicians representing hepatology, nephrology, intensive care, surgery, and pharmacology. The conference was held over 2 days and followed the established ADQI process (http://www.ADQI.org) using a modified Delphi method to achieve consensus.^13^ Conference participants were divided into five working groups. In the pre-conference phase, each group identified a list of key questions and conducted a systematic literature search. During the conference, a series of plenary and breakout sessions were held where work groups developed consensus positions and recommendations that were refined through iterative discussions in plenary sessions. Statements were then proposed and supported by evidence, and by consensus where evidence was limited. The quality of the overall evidence and the strength of recommendations were graded using the GRADE (Grading of Recommendations Assessment, Development and Evaluation) criteria (Table S1).^14–16^ Following the meeting, the contributions of all groups were merged and reconciled by the steering group to generate this conference report following revision and approval by each of the participants.

## Epidemiology and definition of kidney dysfunction in patients with cirrhosis

### How should definitions for AKI, AKD, CKD and renal recovery in patients with cirrhosis be harmonized between kidney disease: Improving global outcomes (KDIGO) and ICA?

#### Consensus statements

• In patients with cirrhosis, we recommend defining AKI using KDIGO criteria: increase in serum creatinine (SCr) ≥0.3 mg/dl (26.5 μmol/L) within 48 h or ≥50% from baseline value known or presumed to have occurred within the prior 7 days and/or urine output (UO) ≤0.5 ml/kg for ≥6 h **(strong recommendation, grade A).**

- In patients with cirrhosis, we recommend defining AKD and CKD using KDIGO criteria **(strong recommendation, grade A).**
- In patients with cirrhosis, we recommend defining complete recovery from AKI as a return of SCr to within 0.3 mg/dl (26.5 μmol/L) of baseline **(strong recommendation, grade B).**

##### Rationale:

**Fig. 1.**
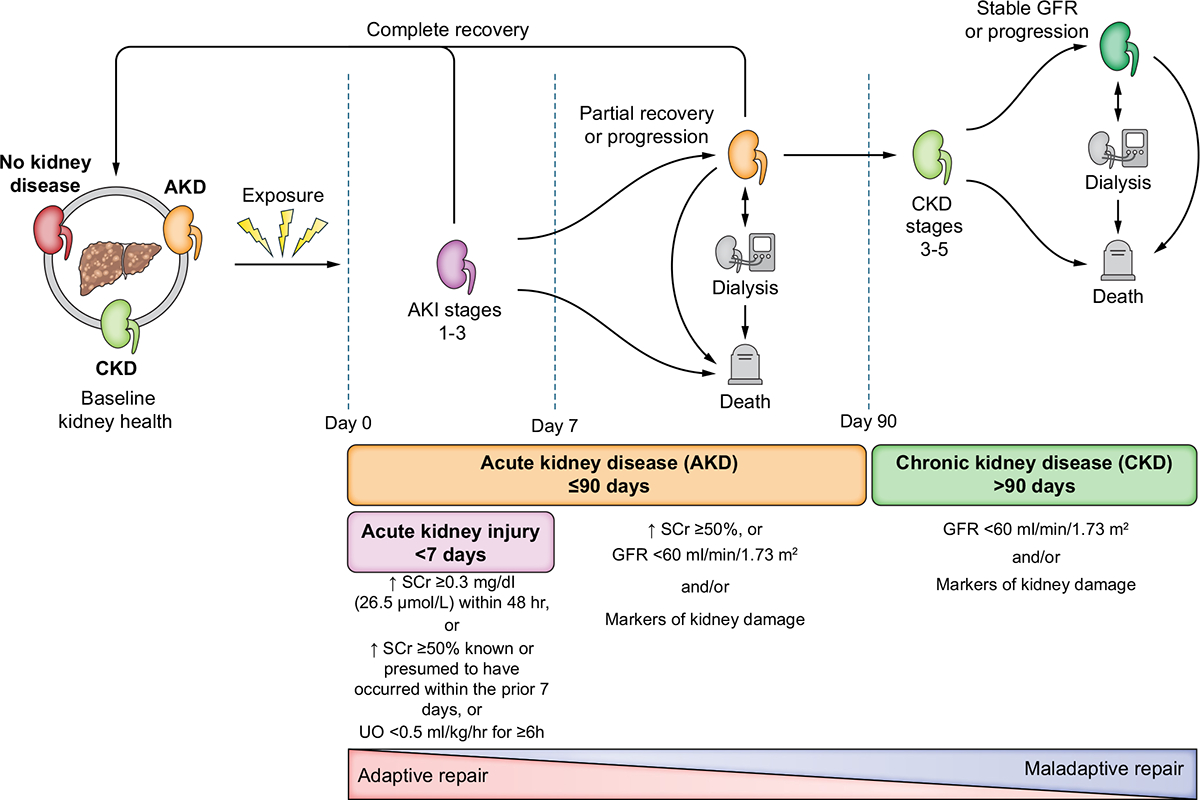
Clinical course and outcomes of AKI in patients with cirrhosis. AKI, AKD and CKD form a continuum whereby initial kidney injury can lead to recovery (adaptive repair), persistent renal injury, and/or eventually CKD (maladaptive repair). Multiple episodes of AKI may occur over the course of an illness within one individual. After AKI resolves, patients may still have abnormalities in kidney function and/or structure that fulfil the criteria for AKD. AKI is a subset of AKD, therefore, all patients with AKI are considered to have AKD. The absence of criteria for AKI, AKD or CKD represents no kidney disease (NKD). Liver or liver-kidney transplantation in select patients may occur at any time. Patients who meet HRS criteria are considered to have HRS-AKI, HRS-AKD or HRS-CKD based on the timing and duration of kidney dysfunction. Patients with HRS-AKD meeting AKI criteria are classified as having HRS-AKI. HRS for less than 90 days would be classified as HRS-AKD, while HRS persisting for more than 90 days would be classified as HRS-CKD. In contrast, a patient with pre-existing CKD (*e.g*., diabetic nephropathy) who develops HRS-AKI would be classified as having HRS-AKI on CKD. AKD, acute kidney disease; AKI, acute kidney injury; CKD, chronic kidney disease; HRS, hepatorenal syndrome. Adapted from Acute Disease Quality Initiative 29, www.ADQI.org, CC BY 2.0 (https://creativecommons.org/licenses/by/2.0/)

AKI, AKD and CKD are classified by KDIGO according to duration and severity of structural and functional abnormalities (Fig. 1).^17^ The ICA currently defines and stages AKI by KDIGO SCr criteria only^11^; however, oliguria is a sensitive and early marker of AKI that is associated with worse outcomes in critically ill patients with cirrhosis.^9^ Most cases of AKI will fulfil both SCr and UO criteria but clinical judgement should be utilised, taking into consideration that UO at baseline may be low in patients with cirrhosis and ascites. Measurement of UO, especially, outside the intensive care unit (ICU) is often inaccurate, and the frequent use of diuretics may affect inter-pretation; however, when possible, close monitoring of UO should be performed in order to detect moderate to severe AKI earlier, and reduce fluid overload.^18^ Recently, a combination of damage and functional biomarkers was proposed by ADQI to be used, along with clinical information, to define AKI and improve diagnostic and staging accuracy,^19^ but their role in patients with cirrhosis remains to be determined. CKD is defined as persistent glomerular filtration rate (GFR) <60 ml/min/1.73 m^2^ and/or markers of kidney damage for >3 months.^17^ Some individuals may have significant abnormalities of structure and/or function (GFR <60 ml/min/1.73 m^2^ or increase in SCr by >50%) within a duration of ≤3 months that do not fulfil the definitions of AKI or CKD; this period is described as AKD (Fig. 1).^20^ AKI is a subset of AKD, therefore, any patient with AKI, by definition, has AKD.

To date, a universal definition of post-AKI renal recovery is not available and remains controversial. Distinct phenotypes based on clinical course have been described in critical illness, defining full recovery as a return of SCr to within 0.3 mg/dl (26.5 μmol/L) of baseline, which also aligns with ADQI^21^ and ICA recommendations.^11,21–23^ However, it is important to appreciate that use of the creatinine criteria may result in overestimation of recovery by ignoring the loss of muscle mass that occurs during critical illness.^24^

### What reference SCr value should be used to define AKI in patients with cirrhosis?

#### Consensus statements

- We recommend using the lowest stable SCr value obtained in the previous 3 months for the diagnosis and staging of AKI. If no values are available in the previous 3 months, the most recent value up to 12 months prior may be used **(strong recommendation, grade D).**
- In the absence of a known baseline SCr, we suggest using the lower of either SCr on admission or SCr back calculated from an estimated GFR (eGFR) of 75 ml/min/1.73 m^2^ as the reference value **(weak recommendation, grade B).**

##### Rationale:

A SCr value is required to diagnose and stage AKI, to evaluate the extent of renal recovery, and to establish a reference point in studies examining the long-term consequences of AKI.^25^ What is considered a baseline SCr remains controversial and is inconsistently defined in the general population, especially in patients without any previous values.^25,26^ The use of known SCr values is superior to imputation,^27^ and therefore, all efforts should be made to identify a prior SCr level, preferably within the previous 3 months.^28^ When more than one SCr value is available, utilising median SCr reduces the biases from outliers and normal physiologic variation and is reliable for estimating baseline kidney function in the general population.^25,27–30^ However, if significant fluctuations exist across multiple SCr values, clinical judgement is crucial to determine the SCr that best reflects the most appropriate baseline value. If no SCr values are available from the prior 3 months, the most recent SCr value up to 12 months prior may be used as a reference SCr, with attention paid to the clinical trajectory to ensure that decrements in kidney function on presentation are truly acute and not due to the presence of progressive CKD.^27^ Thus, it is imperative that all patients with presumed AKI be evaluated for the presence of pre-existing CKD using all available data (*i.e*. clinical history, physical exam, laboratory data, and renal ultrasound).

In the rare instance when no previous baseline SCr values are available, the ICA has suggested the first documented SCr value on hospital admission be used as the reference SCr.^11^ However, this may underestimate the incidence and severity of AKI, and potentially miss the diagnosis of community-acquired AKI, as the SCr may have already increased prior to hospitalization.^9^ In patients with no prior SCr value available, KDIGO recommends the lower value of the admission SCr or SCr derived from eGFR (assuming a baseline GFR of 75 ml/min/1.73 m^2^) be used to decide the reference SCr.^9,17,30,31^ This method was studied in a retrospective study of 3,458 patients with cirrhosis.^9^ The average SCr on the day of admission in patients who developed stage 3 AKI was 1.6 mg/dl, however, an imputed SCr derived from back-calculation was 1.0 mg/dl, a value closer to the known baseline SCr (1.1 mg/dl). There is currently no superior alternative, thus we propose that KDIGO recommendations be followed until a better methodology is verified. The above recommendations for baseline SCr are to facilitate the clinical diagnosis of AKI and should not replace clinical judgement, as AKI remains a clinical diagnosis.

### What are the diagnostic criteria for AKI due to HRS (HRS-AKI)?

#### Consensus statements

- HRS-AKI is a phenotype of AKI that is specific to patients with advanced cirrhosis and ascites; it may also occur in the presence of tubular injury, proteinuria, and/or pre-existing CKD **(not graded).**
- We recommend the following diagnostic criteria for HRS-AKI: a) cirrhosis with ascites; b) increase in SCr ≥0.3 mg/dl (26.5 μmol/L) within 48 h or ≥50% from baseline value, known or presumed, to have occurred within the prior 7 days and/or UO ≤0.5 ml/kg for ≥6 h; c) absence of improvement in SCr and/or UO within 24 h following adequate volume resuscitation (when clinically indicated); and d) absence of strong evidence for an alternative explanation as the primary cause of AKI **(not graded).**
- We recommend against systematic administration of albumin for 48 h as a requisite for the diagnosis of HRS-AKI **(strong recommendation, grade D).**
- We recommend replacing the historical terms HRS type 1 and 2 with the terms HRS-AKI, HRS-AKD and HRS-CKD, depending on the timing and duration of kidney dysfunction **(strong recommendation, grade D).**

##### Rationale:

**Fig. 2.**
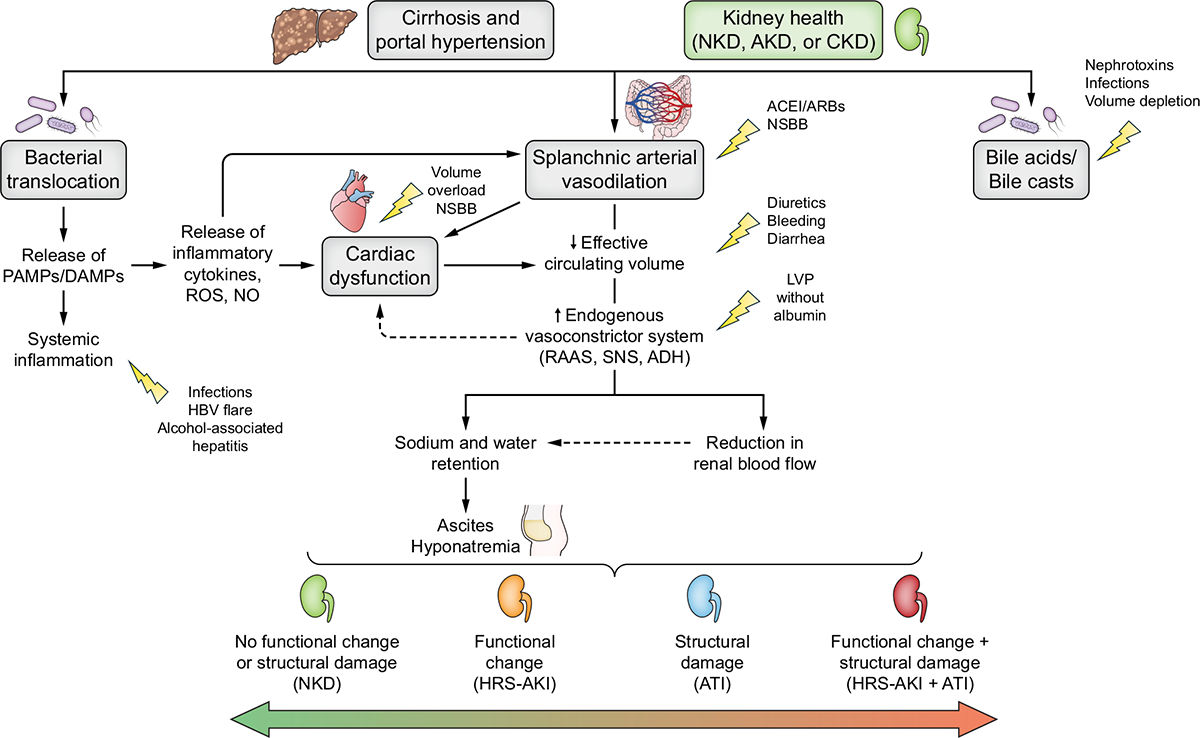
Contemporary concepts in the pathophysiology of AKI. Multiple simultaneous mechanisms can contribute to the development of different phenotypes of AKI in patients with cirrhosis. Background susceptibility to renal injury varies across individuals, according to non-modifiable (*e.g*., comorbidity burden) and modifiable factors (*e.g*., sepsis) and includes liver-related (*e.g*., severity of liver disease, decompensating events), kidney-related (*e.g*., CKD, eGFR), cardiovascular (*e.g*., cirrhotic cardiomyopathy), comorbidities (*e.g*., hypertension, diabetes), and external factors (*e.g*., nephrotoxic drugs, sepsis, excessive diuretics or laxatives). The clinical condition of the liver, kidney, and heart, in addition to concomitant precipitating events and exposures (yellow arrows) may lead to a variety of clinical AKI phenotypes. The different phenotypes of AKI include presence of functional changes (*i.e*. increase serum creatinine and/or cystatin C, and decrease urine output), structural damage (*i.e*. albuminuria, urinary casts, urinary biomarkers) or both. The arrows show progression (red), regression or recovery (green) between the different phenotypes. ACEi, angiotensin converting enzyme inhibitor; ADH, anti-diuretic hormone; AKD, acute kidney disease; AKI, acute kidney injury; ATI, acute tubular injury; ARB, angiotensin receptor blocker; CKD, chronic kidney disease; DAMPs, damage-associated molecular patterns; HBV, hepatitis B virus; LVP, large volume paracentesis; NKD, no kidney disease; NO, nitric oxide; NSBBs, non-selective beta-blockers; PAMPs, pathogen-associated molecular patterns; RAAS, renin-angiotensin-aldosterone system; ROS, reactive oxygen species; SNS, sympathetic nervous system. Adapted from Acute Disease Quality Initiative 29, www.ADQI.org, CC BY 2.0 (https://creativecommons.org/licenses/bi/2.0/)

HRS phenotype describes renal dysfunction in patients with cirrhosis and ascites (a *sine qua non* in the diagnosis of HRS), caused by reduced renal perfusion through haemodynamic alterations in the arterial circulation and overactivity of the endogenous vasoactive systems (Fig. 2).^12,32,33^ Systemic inflammation contributes to neurohumoral and vasodilatory derangements resulting in functional AKI (HRS-AKI) that persists despite adequate fluid resuscitation and may be reversible with vasoconstrictive therapy. In patients with cirrhosis and ascites who present with AKI, HRS-AKI (Box 1) is an essential part of the differential diagnosis and may not always occur in isolation. Even where other aetiologies of AKI coexist, HRS-AKI may be the primary cause of AKI. Therefore, appropriate, and rapid work-up and diagnosis of the cause of AKI are crucial in ensuring timely recognition and treatment of HRS-AKI. Intravascular volume should be assessed^34–36^ in all patients who present with AKI. In those with clinical and haemodynamic evidence of intravascular volume depletion, assessment of response to fluid resuscitation^34–36^ should be completed within 24 h, to ensure early diagnosis and initiation of treatment for HRS-AKI. In patients who are euvolemic or have evidence of intravascular fluid overload, 48 h of albumin infusion for the diagnosis of HRS-AKI is not appropriate and will lead to fluid accumulation. In addition, 48 h of systematic administration of albumin may also delay the initiation of terlipressin in patients who are euvolemic at baseline. Where volume status is equivocal and/or difficult to assess, to exclude any reduction in intravascular volume as the cause of AKI, a fluid challenge (250–500 ml of crystalloid or 1–1.5 g/kg of 20–25% albumin) may be prescribed and, if there is no improvement in SCr and/or UO within 24 h, a diagnosis of HRS-AKI should be considered.

Strong evidence for an alternative explanation such as septic shock requiring vasopressors, acute glomerular injury, obstruction, or nephrotoxin-induced AKI (where an improvement in renal function is expected after withdrawal of drugs) as the *primary* cause of AKI should be sought. Analysis of urinary sediment and damage markers may be useful to detect acute glomerular and/or severe tubular damage, although the thresholds for biomarkers remain to be determined (Table S2). Given the increasing prevalence of metabolic syndrome and diabetes-related kidney disease, isolated proteinuria might be related to comorbidities in the patient and pre-existing CKD and/or proteinuria does not rule out HRS-AKI.

We acknowledge that clinical uncertainty will persist in some cases and whilst enrolment in clinical trials requires that many uncertain cases are excluded (best interest of advancing science), clinical practice mandates that uncertainty is managed in the best interest of the patient. There will be cases where a provisional diagnosis of HRS-AKI is made on the best available evidence and is excluded later as more information becomes available. Since HRS-AKI can coexist with other causes of AKI, patients with co-existing structural damage may still respond to treatment with vasoconstrictors given the presence of altered haemodynamics. Thus, although a lack of HRS-AKI-targeted vasoconstrictor response should trigger re-evaluation for other causes of AKI, non-response does not exclude a coexisting diagnosis of HRS-AKI.

A rapid reduction in kidney function, previously referred to as HRS type-1, is most often precipitated by infections, in particular spontaneous bacterial peritonitis (SBP), however variceal bleed and large volume paracentesis (LVP) without sufficient albumin administration have also been implicated.^37^ Conversely, HRS type-2 was characterised by a slower and more chronic decline in renal function in the setting of refractory ascites. We recommend using the terminology HRS-AKI, HRS-AKD or HRS-CKD based on timing and duration of kidney dysfunction, instead of the historical HRS type-1 and type-2. HRS for less than 90 days would be classified as HRS-AKD, while HRS persisting for more than 90 days would be classified as HRS-CKD. Patients with HRS-AKD meeting AKI criteria are classified as having HRS-AKI. In contrast, a patient with pre-existing CKD (*e.g*., diabetic nephropathy) who develops HRS-AKI would be classified as having HRS-AKI on CKD.

### What is the epidemiology and what are the outcomes of kidney dysfunction in patients with cirrhosis?

#### Consensus statement

- AKI and AKD are common in patients with cirrhosis; prognosis depends on the severity of kidney and liver disease **(not graded).**
- Risk of *de novo* CKD is high following AKI and is associated with worse clinical outcomes **(not graded).**

##### Rationale:

Incidence and outcomes of AKI in patients with cirrhosis vary according to the heterogeneity in severity of illness (both kidney and liver health), the aetiology of AKI, variations in AKI definitions, the diversity of clinical settings and, importantly, inconsistent reporting of outcomes. A diagnosis of AKI (even stage 1 AKI) has been shown to be associated with an increased risk of mortality at 30 days, 90 days and 1 year, compared to no AKI, even following recovery from AKI.^5,9^ Risk factors with the strongest association for developing AKI include CKD, sepsis, SBP, and presence of ascites.^9,38,39^ In-hospital renal replacement therapy (RRT) is required in between 5–47% of patients, with mortality rates between 60–80%.^1,6,40,41^ Independence from RRT is unlikely if not achieved by 3 months post-discharge and occurs in only 26% within 1 year post-discharge.^6,9,42^

The incidence of AKD, defined by KDIGO^43,44^ or as AKI persisting beyond 7 days3, is approximately 30% in patients with cirrhosis, with risk factors including older age, stage 2/3 AKI, CKD, diabetes, ascites, infection and community-acquired AKI.^3,43^ The prevalence of CKD in patients with cirrhosis has increased over the years, probably owing to increased recognition coupled with the increased prevalence of metabolic risk factors.^3,45^ The transition from AKI or AKD to CKD is poorly described in patients with cirrhosis, but emerging data suggest that the risk of developing *de novo* CKD is high in AKI survivors, occurring in 14–25% of patients, and is associated with worse clinical outcomes including increased risk of hospital readmission, further episodes of AKI, refractory ascites, and bacterial infections during follow-up.^33,43,45^

## Pathophysiology of AKI in patients with cirrhosis

The degree of liver, kidney, and cardiac derangement, together with concomitant precipitating events and exposures may lead to a variety of clinical phenotypes of AKI (Fig. 2).^12^ Susceptibility to AKI follows development of portal hypertension through increased intrahepatic resistance from liver fibrosis and vasodilation of splanchnic vascular beds secondary to bacterial translocation and systemic inflammation. Vasodilatation leads to a decrease in effective central blood volume that, in turn, leads to activation of sodium/water conservation and vasoconstrictive neurohumoral pathways. Progression of cirrhosis and portal hypertension leads to further vasodilatation and consequently increased activation of these neurohumoral systems, leading to ascites, extreme renal vasoconstriction and HRS-AKI.

Cardiac dysfunction may contribute to AKI development although the mechanisms are controversial. In the early phase of decompensated cirrhosis, the cardiac output (CO) increases but release of cardio-depressive substances leads to subclinical changes in the myocardium^46^ and impairment of cardiovascular reflexes which, coupled with cardio-depression and diastolic dysfunction, is termed ”cirrhotic cardiomyopathy”.^47^ Small cohort studies have suggested that a relative reduction of CO results in renal hypoperfusion and might predict the development of HRS-AKI.^48,49^ Use of non-selective beta-blockers to prevent variceal bleeding has been associated with a greater risk of developing HRS-AKI and to increased mortality in selected patients with refractory ascites and documented inappropriate CO.^50–53^ However, two recent studies demonstrated significantly higher CO in patients with HRS-AKI compared to those without.^54,55^ Consequently, the predominant pathophysiological mechanism behind HRS-AKI may not be directly related to reduced CO but rather driven by an inability to increase CO in response to stress, a hallmark of cirrhotic cardiomyopathy.^55^ Collectively these seemingly disparate findings suggest that perhaps there is a “window” during the development of HRS-AKI in which impaired cardiac response to stress leads to a low CO. Interventions which worsen this trajectory (*e.g*., non-selective beta-blockers, un-guided volume expansion) may in fact impede renal recovery. However, whether interventions that protect or improve CO result in improved renal function is currently unknown.^56^

Systemic inflammation is common in patients with decompensated cirrhosis (Fig. 2).^57,58^ Bacterial/bacterial product translocation and/or overt infection, which is associated with release of PAMPs (pathogen-associated molecular patterns), are fundamental in the development of HRS-AKI, particularly in patients with acute-on-chronic liver failure (ACLF). PAMPs activate innate host immunity, and release of proinflammatory cytokines, vasodilators and reactive oxygen species which may all impair renal function.^59,60^ Renal tubular Toll-like receptor 4 is also upregulated in patients with AKI, likely through bacterial translocation.^61^

The toxic effect of bile acids on tubular cells has been documented and the mechanisms leading to toxicity have been demonstrated recently in animal models.^62^ However, in the absence of diagnostic tests, the thresholds of bile acids and serum bilirubin associated with AKI in patients with severe cholestasis remain largely unknown.^63^

### What are the determinants of susceptibility and trajectory for AKI and its recovery in patients with cirrhosis?

#### Consensus statement

- Modifiable and non-modifiable factors affect susceptibility to AKI and determine the severity as well as the trajectory of recovery **(not graded).**

##### Rationale:

Background susceptibility to AKI varies across individuals according to liver- (*e.g*. severity of liver disease, ACLF, decompensating events) and kidney-related factors (CKD, baseline kidney function), cardiovascular status (*e.g*. cirrhotic cardiomyopathy), concurrent comorbidities (*e.g*. hypertension, diabetes), and external elements which may be either modifiable (*e.g*., presence of infection, liver disease aetiology, nephrotoxins, volume depletion) or non-modifiable (*e.g*., comorbidity burden). The trajectory of post-AKI recovery is influenced by resolution of the precipitating events, the aetiology and severity of AKI, presence of underlying CKD, renal reserve, the severity of liver disease, degree of adaptative and maladaptive repair, and regenerative mechanisms.^64^ Adaptive repair is characterised by tubular proliferation, repair and regeneration of endothelial cells, which leads to resolution and return to normal kidney structure.^65^ Maladaptive repair is characterised by fibrosis, tubular loss and delayed resolution of inflammation with subsequent loss of functional renal reserve and has been shown to play a central role in the transition from AKI to CKD.^65^ Factors associated with a switch from adaptive to maladaptive repair are thought to include advanced age, AKI phenotype, severity, duration and frequency of injury, and baseline kidney health.^66^

Patients with decompensated cirrhosis are prone to develop repeated episodes of AKI following sepsis, hypovolemia and circulatory changes associated with LVP and may develop irreversible chronic kidney changes. While no data exist on maladaptive repair in HRS-AKI, there is recognition that HRS-AKI may not be an entirely functional entity due purely to haemodynamic derangements. Patients with intense renal vasoconstriction and systemic inflammation (as seen in HRS-AKI) may have sustained kidney hypoxia, resulting in concomitant acute tubular injury (ATI), as demonstrated on kidney biopsy findings of patients with HRS^67,68^ and by the overlap in biomarkers in patients with HRS-AKI and ATI.^69–73^

## Prevention and work-up of AKI in patients with cirrhosis

### What are the approaches for prevention of AKI in patients with cirrhosis?

#### Consensus statements

- We recommend strategies to mitigate the risk of AKI that include a personalised kidney-liver health (KLH) assessment to inform susceptibility to AKI, nephrotoxin stewardship, and liver-specific recommendations for anticipated and unanticipated exposures **(best practice statement).**
- We recommend 20–25% albumin for the prevention of AKI following LVP and in patients with SBP **(strong recommendation, grade B).** The dose and duration of albumin administration should be guided by patients’ haemodynamic and volume status **(best practice statement).**
- We recommend against the systematic use of albumin in patients with decompensated cirrhosis for a) the prevention of AKI in patients with non-SBP infections, and b) solely to maintain a serum albumin concentration >3.0 g/dl **(strong recommendation, grade A).**

##### Rationale:

**Fig 3.**
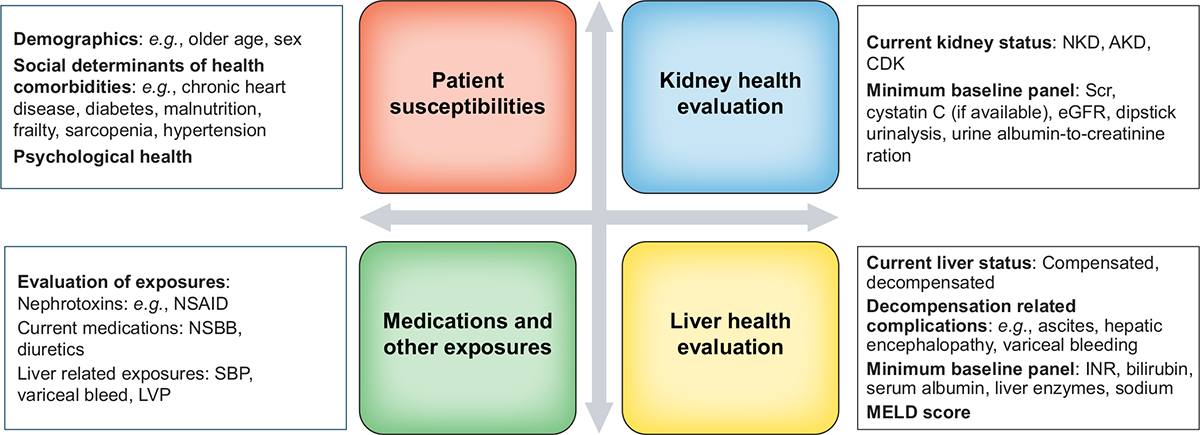
Kidney-liver health assessment. Kidney-liver health assessment is a ‘living’ process that should be repeated if the patient’s condition changes and following planned or unplanned exposure, both during hospitalization and post-AKI care in the outpatient setting. AKD, acute kidney disease; AKI, acute kidney injury; CKD, chronic kidney disease; eGFR, estimated glomerular filtration rate; INR, international normalised ratio; MELD, model for end-stage liver disease; NKD, no kidney disease; NSAID, non-steroidal anti-inflammatory drug; NSBBs, non-selective beta-blockers. Adapted from Acute Disease Quality Initiative 29, www.ADQI.org, CC BY 2.9 (https://creativecommons.org/licenses/by/2.0/)

A comprehensive KLH assessment offers opportunities for surveillance measures and targeted prevention strategies, both before an anticipated exposure and following an AKI-inducing event (Fig. 3).^74^ Prevention of AKI in patients with cirrhosis includes general measures that apply to all patients at risk of AKI,^17^ as well as those unique to patients with cirrhosis (Table 1).^75–77^ Nephrotoxin stewardship entails assessment of potential exposure, surveillance for drug-related events and ensuring safe medication use.^78–80^ Approximately 30% of patients with cirrhosis experience a potentially avoidable adverse drug event.^81^ Drug dosing can be particularly challenging in patients with cirrhosis as relatively lower SCr concentrations may lead to overestimation of GFR.^82^

The role of intravenous albumin in the prevention of AKI has been studied in several randomised-controlled trials (RCTs) (Table S3). In patients with SBP, treatment with antibiotics in addition to 20% albumin administration (at an arbitrary dose of 1.5 g/kg on day 1 and 1.0 g/kg on day 3) has been associated with lower rates of AKI and mortality compared to antibiotics alone.^83^ However, this benefit has only been demonstrated in patients with serum bilirubin >4 mg/dl or SCr >1.0 mg/dl.^83–85^ Administration of albumin should consider the patient’s haemodynamic and volume status. Whether all patients with SBP should receive routine albumin administration, or the optimal dose^86^ and duration of albumin treatment for the prevention of AKI in patients with SBP, remain to be determined. Systematic administration of albumin in hospitalized patients with non-SBP infection^87–90^ or the use of daily albumin to target an albumin level >3.0 g/dl^91^ have been associated with higher risk of pulmonary oedema with no effect on AKI incidence or survival. RCTs on the long-term administration of 20–25% albumin in the outpatient setting in patients with uncomplicated ascites have led to conflicting results.^92,93^ The lack of survival benefit in MACHT may be because few patients completed the 12-month follow-up (10% in those receiving albumin and 20% in the placebo group), as many underwent liver transplantation (LT).^93^ Meanwhile, in the ANSWER trial, patients receiving albumin were seen more frequently compared to those in the control group, thus the observed survival benefit could have resulted from earlier detection and treatment of complications.^92^ Therefore, there is insufficient evidence to recommend long-term outpatient administration of albumin for the prevention of AKI in patients with uncomplicated ascites.

Compared to alternative treatments, administration of 20–25% albumin (6–8 g for every litre over 5 L of ascites removed) during LVP is associated with lower incidence of post-paracentesis circulatory dysfunction, a known trigger for AKI, specifically HRS-AKI.^94,95^ In patients with refractory ascites, transjugular intrahepatic portosystemic shunt (TIPS) has been shown to be effective at controlling ascites and may thereby prevent the development of HRS-AKI.^96^ An implantable medical device, alfapump^®^ (Sequana Medical NV, Ghent, Belgium) enables mobilization of ascitic fluid to the bladder for urinary excretion and has been shown to reduce the frequency of LVPs in patients with refractory ascites. However, it is not widely available and has been associated with AKI if the volume of ascites removed early after insertion is high, thus regular administration of albumin may be required to prevent AKI.^97–100^

### What diagnostic tools should be included in the work-up of patients with cirrhosis and AKI?

#### Consensus statements

- We recommend using similar tools for the diagnostic work-up for AKI in patients with cirrhosis as used in those without cirrhosis **(best practice statement).**
- We suggest using the Chronic Kidney Disease Epidemiology Collaboration (CKD-EPI) eGFR equation without the race variable, and preferably with cystatin C (CysC), for assessment of kidney function, though the performance at low GFR and in those with ascites may be suboptimal **(weak recommendation, grade B).**
- In addition to SCr, we suggest complementary use of functional and damage-related markers to aid in timely detection of AKI, characterisation of different AKI phenotypes and to guide treatment strategies **(weak recommendation, grade B).**

##### Rationale:

The diagnostic evaluation of patients with cirrhosis and AKI includes clinical history, assessment of intravascular volume status, and detection of potential precipitants. Assessment of intravascular volume remains challenging as most currently available haemodynamic monitoring tools have not been studied in patients with cirrhosis.^34,35^ Point-of-care ultrasonography has been suggested as a tool to assess volume status at the bedside; however, it is prone to interobserver variability and is challenging to use in patients with significant ascites.^101–103^ Examination of urinary sediment is difficult in patients with elevated bilirubin levels due to staining of cells and casts. Additionally, significant interobserver variability and discordance with kidney biopsy have been reported.^68,104,105^ Complications from percutaneous renal biopsy are documented in up to 30% of cases compared to 0.9% in the general population;^106,107^ however, low complication rates have been reported using the transvenous route, even in patients with coagulation disorders.^68,105,108^

###### Assessment of kidney function

Diagnosis of AKI may be missed or delayed in patients with cirrhosis given SCr is influenced by reduced muscle mass, increased volume of distribution in the setting of fluid overload,^109^ and interference with bilirubin.^110^ SCr may also be falsely lowered by large volume blood transfusions. CysC allows for earlier diagnosis of AKI in patients with cirrhosis with rising levels often preceding changes in SCr by 48 h, and is a useful prognostic marker for renal outcomes and mortality.^111–115^ In a large prospective study in patients with cirrhosis, the addition of CysC to the components of the model for end-stage liver disease (MELD) score was superior to MELD for prediction of overall mortality.^111^ In addition, CysC provides a better estimation of renal function, especially in patients with prolonged critical illness, and may help in drug dosing and management of nephrotoxic drugs.^24,116^

eGFR equations, such as MDRD or CKD-EPI equations, were developed and validated in patients with CKD and are inaccurate for the assessment of renal function in patients with AKI, as they require SCr to be in a ‘steady state’. eGFR is one of the factors used to determine candidacy for simultaneous liver and kidney transplantation (SLKT), yet current equations tend to overestimate the true GFR by 10 to 20 ml/min/1.73 m^2^, especially in those with a GFR <40 ml/min/1.73 m^2^, ascites, or both.^117,118^ In patients with cirrhosis with a GFR <60 ml/min/1.73 m^2^, use of the CKD-EPI-CysC eGFR equation demonstrated the least bias (overestimated GFR by 10.3 ml/min/1.73 m^2^) with acceptable precision and accuracy.^117^ Thus, efforts to enable increased, routine and timely use of CysC, especially to confirm eGFR in patients who are at risk of or have CKD, should be undertaken as this may also allow clinicians to better identify candidates for SLKT.^26,117,119^ Recently, several eGFR equations were developed specifically in patients with cirrhosis to allow for more accurate GFR estimation in this patient population.^120–122^ In 2021, a new CKD-EPI equation, which included the removal of race as a variable, was introduced and widely implemented in the US as an important step in efforts to eliminate disparities in the care of patients with kidney disease; however, this equation has not been widely adopted outside the US.^119,123^ Preliminary data suggest acceptable performance in patients with cirrhosis, though their role in patients with low GFR and ascites remains to be studied.^124^ A meta-analysis of studies on timed urine collection for GFR estimation by creatinine clearance in patients with cirrhosis demonstrated overestimation of true GFR, especially in those with low GFR (<60 ml/min/1.73 m^2^).^125^ In patients without cirrhosis, the composite of timed urinary urea clearance and creatinine clearance (former tends to underestimate, and the latter overestimate, the true GFR) showed superior performance over CKD-EPI equations and creatinine clearance alone when compared to measured GFR, especially in patients with GFR <60 ml/min/1.73 m^2126^; however, this has not been studied in patients with cirrhosis.

###### AKI phenotyping: Role of biomarkers

**Fig. 4.**
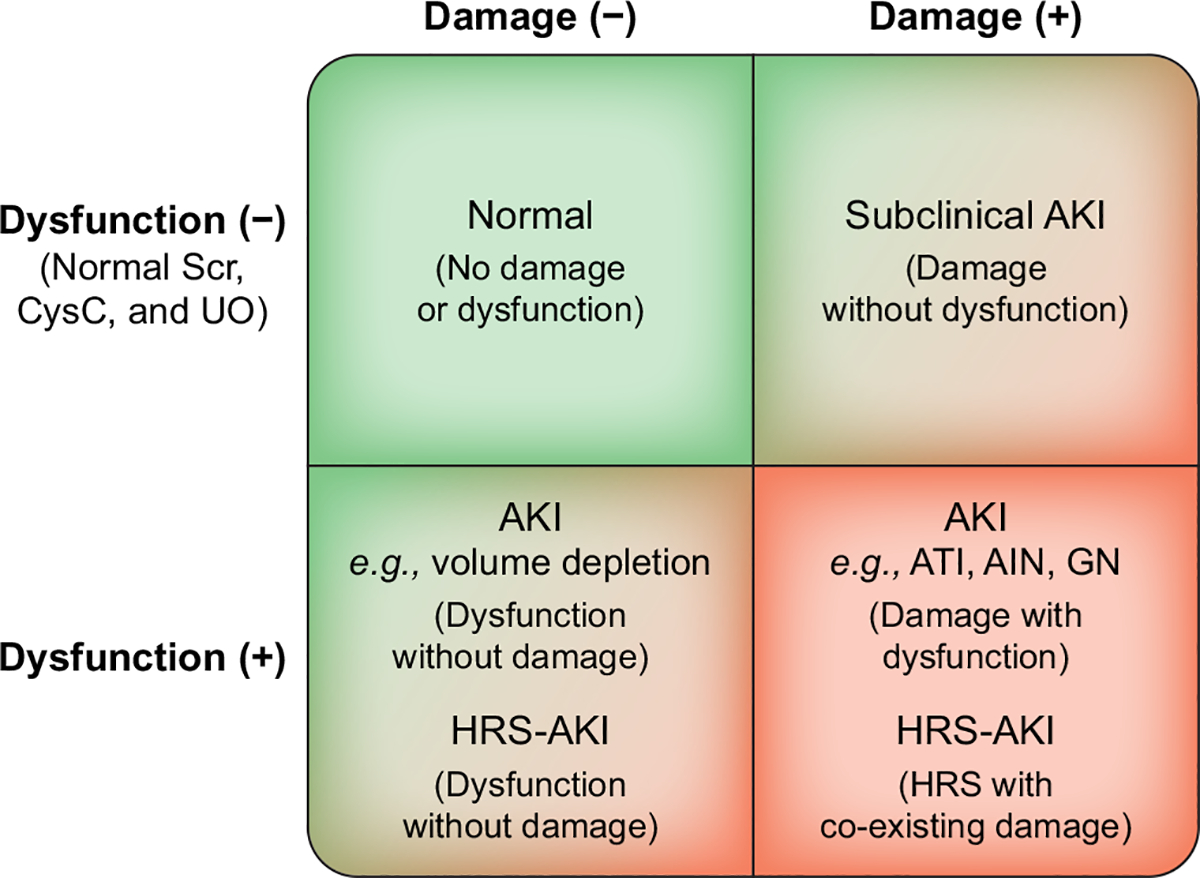
Proposed framework for evaluating AKI phenotypes based on combination of functional and damage markers. At any given point in time, patients would fall into one of the four quadrants, based on the results of the representative functional and damage marker tests and could be assessed over time to see their transitions across the categories. The ability to detect a state of damage alone (right upper quadrant) represents a “subclinical” state from which loss of function might develop after several days or not at all. Markers of kidney damage may include albuminuria/proteinuria, hematuria, urinary casts, and biomarkers. Bottom left quadrant indicates an acute change in kidney filtration but without detectable kidney damage such as seen in patients with volume depletion. Patients who meet criteria for HRS may be either without evidence of damage (left lower quadrant) or have co-existing damage (right lower quadrant). Sequential assessments could provide information on which of the factors is prevalent for ongoing injury or resolution and offer opportunities for targeted intervention. It is expected that the process is dynamic, and patients may move from one phenotype to another during the course of their illness. Modified, with permission, from Acute Disease Quality Initiative 10, www.ADQI.org. AIN, acute interstitial nephritis; AKI, acute kidney injury; ATI, acute tubular injury; GN, glomerulonephritis; HRS, hepatorenal syndrome. Adapted from Acute Disease Quality Initiative 29, www.ADQI.org, CC BY 2.0 (https://creativecommons.org/licenses/by/2.0/)

The combined use of functional (*e.g*., Scr, CysC) and damage (*e.g*., albuminuria, urinary neutrophil gelatinase-associated lipocalin [uNGAL]) biomarkers enables more accurate differential diagnosis of the aetiology and mechanisms of AKI in patients with cirrhosis and potentially enables the identification of AKI sub-phenotypes suitable for specific therapeutic interventions (Fig. 4).^127^ Biomarkers may also help to detect those at risk of AKI in whom interventions may limit renal damage.^113,128,129^ However, in the absence of a detectable SCr rise (*i.e*., subclinical AKI), more data are required to define context-specific thresholds for damage-related markers that could act as precise diagnostic criteria for AKI. As further damage and functional biomarkers are discovered and qualified, we believe incorporating them into the proposed conceptual framework (Fig. 4) is an important step towards improving our understanding of the mechanisms and pathophysiology of AKI in patients with cirrhosis (Fig. 2), refining the determination of prognosis and selecting time points and targets for interventions.^127,130–132^

Various markers have been assessed in patients with cirrhosis (Table S2).^71,133,134^ Measurement of the fractional excretion of sodium (FENa) to differentiate ATI from HRS-AKI has been thought to be unhelpful since FENa <1% is common in patients with cirrhosis, even in the absence of AKI.^70,135^ However, if using a lower threshold of FENa of <0.1–0.2% (which may not be possible as many laboratories do not report urine sodium values <20 mEq/L) in combination with other urinary biomarkers and clinical judgement, the test may have improved specificity in identifying HRS-AKI.^70,72^ uNGAL is one of the most promising and widely studied injury biomarkers, with levels significantly increasing in a stepwise manner from HRS-AKI to ATI.^70–73,133,136,137^ A uNGAL value of ~220–250 μg/g creatinine (Bioporto Diagnostics, Hellerup, Denmark) has been demonstrated to distinguish patients with ATI from other phenotypes,^69,72^ with response rates to terlipressin seen in 70% of patients with uNGAL <220 μg/g of creatinine compared to only 33% in those with uNGAL >220 μg/g of creatinine.^136^ Of note, these studies have shown overlap between different phenotypes, which may be due to a combination of patient population heterogeneity, presence of underlying CKD, differences in assays used, results based on adjudicated gold standards rather than histopathological diagnosis, or reflecting possible progression along a continuum from functional to structural causes of AKI. Combining markers such as urinary kidney injury molecule-1, uNGAL, and CysC was better than using one marker alone in identifying HRS-AKI, especially after adding clinical parameters.^138^ Whether the target level of uNGAL that would differentiate between the AKI phenotypes, and/or response to terlipressin would be different with the new diagnostic criteria for HRS-AKI set forth by the authors remains to be determined.

## Management of AKI in patients with cirrhosis

### What strategies are applicable to the management of AKI in patients with cirrhosis?

#### Consensus statements

- We recommend personalised strategies for the management of AKI based on the individual patient’s kidney-liver health profile and AKI phenotype **(best practice statement).**
- We recommend a combination of physical examination, imaging studies, and static and dynamic measurements to guide fluid management, with frequent reassessment throughout all phases of treatment to avoid volume overload **(best practice statement).**
- We recommend crystalloids, preferentially balanced solutions, as first-line therapy for patients with AKI requiring fluid resuscitation, unless a specific indication exists for the use of other fluids **(strong recommendation, grade B).**
- We recommend discontinuation of all fluids and initiation of diuretic therapy or RRT in patients with AKI who demonstrate signs or symptoms of volume overload **(best practice statement).**
- There is insufficient evidence to support routine measurement of intra-abdominal pressure (IAP) in patients with tense ascites and AKI **(not graded).**
- We recommend initiation of RRT be individualised, with consideration of clinical context and anticipated or observed life-threatening AKI-related complications **(best practice statement).**
- We recommend expedited evaluation for LT in patients with decompensated cirrhosis following an episode of AKI **(best practice statement).**
- There is insufficient evidence to recommend TIPS or extracorporeal liver support for the treatment of AKI **(not graded).**

##### Rationale:

Initial management of patients with AKI should follow KDIGO consensus recommendations for AKI, as well as specific guidelines for patients with cirrhosis, which include discontinuation and/or avoidance of nephrotoxins, and optimisation of haemodynamic and volume status.^75–77^ Fluid administration requires careful titration based on severity of kidney disease, degree of oliguria, and phase of resuscitation.^34–36^ Assessment of fluid responsiveness should include careful history and physical examination, vital signs, and a combination of available variables including imaging studies, as well as static and dynamic measurements.^34–36^ No study has demonstrated superiority of a particular method, and therefore the choice of tool depends on the patient’s location (ICU *vs*. general ward) and clinical discretion. Repeated assessment of volume status and close monitoring of UO should be undertaken so that complications of iatrogenic volume overload can be prevented.

Fluid choice should be individualised and guided by specific patient condition: blood products in cases of gastrointestinal bleeding, crystalloids (preferentially balanced solutions such as lactated ringers or PlasmaLyte) in cases of volume depletion, and 20–25% albumin in those with SBP or HRS-AKI, with close attention to patient haemodynamics and volume status.^11,34,35,75,77^ Albumin is often used with the notion that it is more likely to maintain oncotic pressure than crystalloids; however, numerous RCTs in critically ill patients have failed to demonstrate any difference in 30-day or 90-day mortality or need for RRT between groups.^139,140^ In patients with advanced cirrhosis, not only does serum albumin concentration decrease, but its structure and anti-oxidant functions are also altered, reducing its capacity to bind to bacterial products and reactive oxygen species, potentially exacerbating systemic inflammation.^141^ Experimental studies suggest that infusion of normal “exogenous” albumin has beneficial effects on controlling systemic inflammation and improving circulatory status, which could also contribute to the prevention or reversal of AKI; however, this effect has not been observed in clinical practice.^142^ Results from two RCTs comparing albumin to crystalloids in patients with cirrhosis and sepsis-induced hypotension have been conflicting, which may be explained by differences in type of albumin solution (5% *vs*. 20%), type of crystalloid (0.9% saline *vs*. plasmalyte), and the short duration of the studies (7 *vs*. 28 days) (Table S3).^143,144^ Although the use of albumin was associated with a significantly greater improvement in haemodynamics in the short term, the response was not sustained and did not improve renal outcomes or need for RRT compared to crystalloids.^143,144^

Interactions between ascites, IAP, and AKI are complex. In theory, IAP and intra-abdominal compartment syndrome related to large volume ascites may induce AKI by increasing central venous pressure and reducing right ventricular output, and thus CO.^95,145^ In critically ill patients with HRS and tense ascites, paracentesis plus albumin infusion resulted in an increase in creatinine clearance, which correlated with the decrease in IAP.^145,146^ However, LVP which reduces IAP is also known to trigger circulatory changes that may contribute to impaired kidney function without the use of albumin.^147^ Currently, there is insufficient evidence to support routine measurement of IAP in patients with tense ascites and no evidence to support systematic LVP in patients with increased IAP.^148,149^

###### Renal replacement therapy

Recent RCTs have not shown a benefit of accelerated initiation of RRT in critically ill patients; however, patients with cirrhosis were either excluded or largely underrepresented.^150–152^ The timing of RRT in patients with cirrhosis should be individualised, taking into account the trajectory of both kidney and liver health and be considered before overt complications have developed.^17,35,153–155^ Early initiation of RRT should be considered in patients with signs or symptoms of intravascular volume overload without adequate response to diuretics (even in the absence of AKI) or in those in whom volume overload cannot be corrected without serious adverse effects.^35,155^ Patients with cirrhosis and AKI-related metabolic changes are prone to develop encephalopathy and, uremic symptoms can often overlap with hepatic encephalopathy. As such, initiation of RRT should be considered earlier, especially if encephalopathy persists despite treatment. Choice of RRT modality depends on availability, resources, and inherent risks with intervention.^154^ Among patients listed or undergoing evaluation for LT, initiation of RRT should be viewed as a tool to optimise a patient’s condition and as a bridge to LT. For those who are not candidates for LT, we recommend discussion with the patient and/or caregivers regarding goals of therapy, and the poor long-term prognosis, as transplant-free survival is extremely low, especially in those with very high MELD scores.^40,156,157^

###### Transplantation

Episodes of AKI are associated with a high risk of short-term mortality, especially in patients with high MELD scores, and therefore patients may benefit from an expedited inpatient transplant evaluation.^156,158^ Predicting the severity and duration of kidney dysfunction that results in non-recovery of renal function following LT remains a challenge.^159–161^ Current US policies for SLKT incorporate duration of AKI (eGFR ≤25 ml/min for ≥6 weeks, with or without dialysis) and CKD at the time of transplant and introduced a safety net approach which guaranteed prioritisation of kidney transplantation in patients with an eGFR ≤20 ml/min within 1 year following LT.^162^ However, factors such as aetiology of AKI,^163^ older age, and comorbidities (such as diabetes) known to impact post-transplant renal recovery are not included. Biomarkers predictive of AKI recovery after LT could enhance decision-making algorithms regarding the need for SLK.^164,165^ Kidney transplant alone in patients on chronic dialysis may be a feasible option among selected patients with compensated cirrhosis without clinically significant portal hypertension, especially in the setting of a treatable aetiology of liver disease.^166^

###### Transjugular intrahepatic portosystemic shunt

While TIPS placement has been shown to improve GFR over time in patients with refractory ascites, a complication of portal hypertension that shares its pathophysiology with HRS-AKI, it has been studied only sparingly as a treatment for HRS-AKI.^167–171^ A RCT examining TIPS for the treatment of HRS-AKI is currently underway.^172^

###### Extracorporeal liver support

Extracorporeal liver support such as adsorbent columns, albumin dialysis, and plasma exchange have been investigated for use in ACLF and treatment of HE, but not as AKI-specific therapies.^173,174^ Treatment of AKI in patients with ACLF may require targeting not only removal of known substances, such as creatinine and urea, but also removal of a wide spectrum of pathogenic factors and mediators of inflammatory response that are implicated in the pathophysiology of ACLF.^175–177^

### What strategies are specific to the management of HRS-AKI?

#### Consensus statements

- We recommend initiating vasoconstrictor therapy (terlipressin as first-line agent), in combination with 20–25% albumin, immediately upon establishing a diagnosis of HRS-AKI **(strong recommendation, grade A).**
- We recommend close monitoring of volume status during treatment for HRS-AKI. The dose of albumin should be adjusted daily based on patients’ volume status, with immediate discontinuation of albumin if there is evidence of volume overload **(best practice statement).**
- We recommend increasing the dose of terlipressin every 24 h if SCr has not decreased by 25% from baseline **(strong recommendation, grade D)** and increasing the dose of norepinephrine every 4 h if MAP has not increased by ≥10 mmHg from baseline **(strong recommendation, grade B).**
- We recommend discontinuation of vasoconstrictors for HRS-AKI if (a) SCr returns to within 0.3 mg/dl of baseline; (b) a severe adverse reaction develops; (c) kidney function does not improve after 48 h on maximum tolerated doses; (d) RRT is indicated; or (e) maximum of 14 days of therapy **(strong recommendation, grade B).**
- We recommend LT, in select patients, as the definitive treatment for HRS-AKI regardless of response to vasoconstrictor-directed therapy **(strong recommendation, grade A).**

##### Rationale:

Terlipressin is the most studied and consistently effective vasoconstrictor for the treatment of HRS-AKI and its use (preferably as a continuous infusion)^178^ is recommended as a first-line agent (Table 2).^11,75–77^ Meta-analysis and systematic reviews have shown norepinephrine to have comparative effects to terlipressin for reversal of HRS-AKI, with the exception of one study in patients with ACLF wherein terlipressin was demonstrated to be superior.^179–182^ If terlipressin is not available or contraindicated, treatment with norepinephrine may be more appropriate than an initial trial with midodrine and octreotide.^183–185^ However, norepinephrine requires ICU admission and placement of a central venous catheter for continuous infusion.

Current guidelines recommend daily use of 20–25% albumin (20–40 g/day) during the treatment of HRS-AKI; however, the optimal dosing of albumin and length of administration are not well defined. Cautious use of albumin is recommended, with discontinuation if there is evidence of pulmonary oedema.

Clinical trials examining the efficacy of vasoconstrictors for HRS-AKI have used historical definitions of HRS (*i.e*., type-1 HRS) and demonstrated that vasoconstrictors are more effective at improving renal function when initiated at SCr <2.25 mg/dl and when achieving an increase of MAP ≥15 mmHg.^185–188^ Re-evaluation for alternative causes of AKI should be considered if there is an increase in MAP ≥15 mmHg from baseline during vasoconstrictor treatment for HRS-AKI without improvement in SCr. What MAP goal (absolute value or an increase in MAP from baseline) should be targeted during treatment with vasoconstrictors requires further investigation. Whether the current definitions set forth here by the authors will improve the rates of HRS-AKI reversal remains to be determined.

**Fig. 5.**
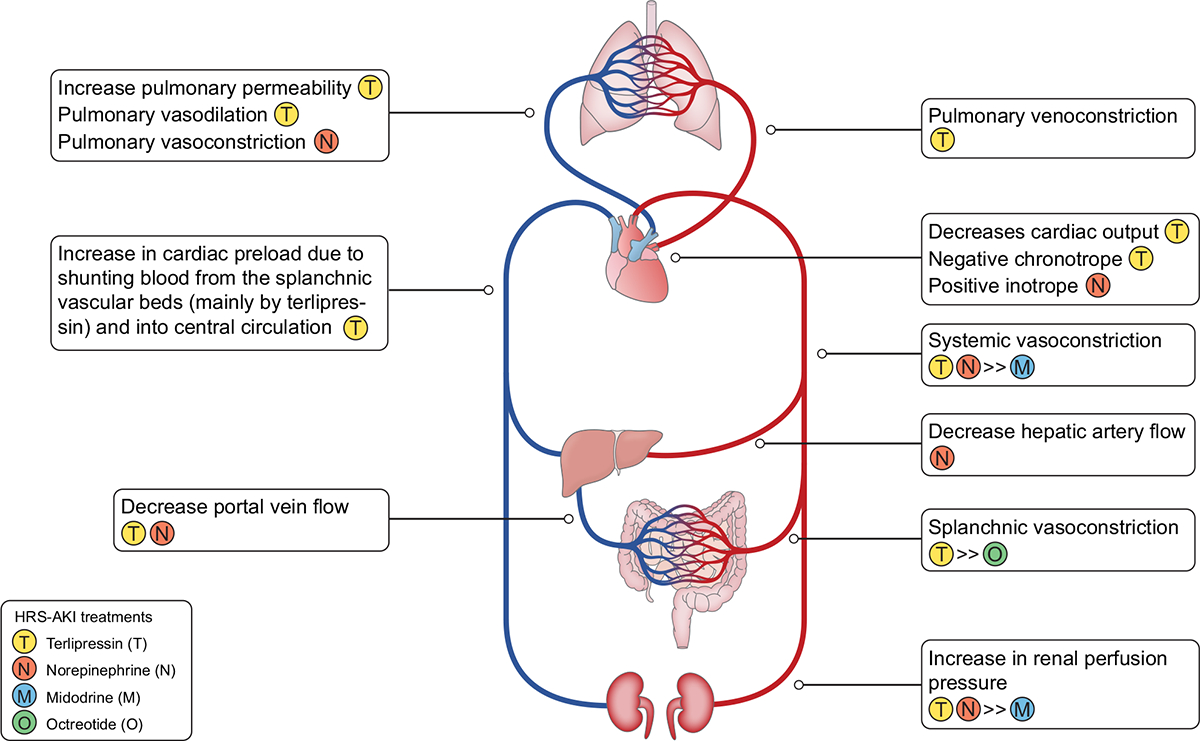
Differential effects of various HRS-AKI treatments on vascular beds, cardiac function, and renal perfusion, as well as pulmonary effects. Terlipressin (T) increases renal perfusion pressure but also decreases cardiac output. By increasing cardiac preload (through shunting of splanchnic blood to central blood), increasing cardiac afterload (due to increase in systemic vascular resistance), and effecting pulmonary vasculature^231–234^ (pulmonary artery dilation, pulmonary vein constriction, as well as possibly an increase in pulmonary capillary permeability), when combined with large doses of albumin, may be associated with an increased incidence of pulmonary oedema. Norepinephrine (N) has a positive inotropic effect and causes systemic vasoconstriction, which then also increases renal perfusion pressure. In contrast to terlipressin, norepinephrine constricts pulmonary arteries without any effect on the pulmonary vein. Midodrine (M) causes weak systemic vasoconstriction and octreotide (O) causes temporary splanchnic vasoconstriction, effects that lead to an only modest increase in renal perfusion. Adapted from Acute Disease Quality Initiative 29, www.ADQI.org/ CC BY 2.0 (https://creativecommons.org/licenses/by/2.0/)

Patients receiving vasoconstrictors should be monitored for adverse events (mainly ischaemic) which are usually mitigated by drug discontinuation, lowering the dose, or in the case of terlipressin, changing from bolus dosing to continuous infusion.^178^ Higher incidence of pulmonary oedema has been reported in patients receiving terlipressin compared to placebo,^189–192^ which may be related to a combination of several pathways in an already critically ill patient population (Fig. 5).^193,194^ Cautious use of terlipressin is recommended in patients with evidence of volume overload, and temporary suspension of albumin together with administration of diuretics may prevent complete discontinuation of vasoconstrictor treatment.^190^

Reversal of HRS-AKI reduces the risk of CKD and need for RRT after LT^195^; however, there is no improvement in transplant-free survival, thus the use of vasoconstrictors should be seen as a bridge to transplantation or renal recovery, rather than a definitive cure. Pharmacological treatment of HRS-AKI lowers the MELD score by lowering SCr, affecting waiting list priority. This can be detrimental for patients awaiting LT in regions with long waiting times and higher average MELD scores at time of transplant, especially when MELD score is updated at very close intervals.^156,192^ Some countries partially mitigate this issue by “holding” the SCr at its apex after the initiation of a vasoconstrictor or assigning extra points for those treated for HRS-AKI, regardless of treatment response, to ensure patients who are treated are not disadvanged.^196^ As new treatments and prognostic scores become available, revisions to transplant allocation policies will be needed to best serve this high-risk patient population.

## Post-AKI/AKD outpatient follow-up in patients with cirrhosis

### What are the key elements of an appropriate post-AKI/AKD care bundle following hospital discharge in patients with cirrhosis?

#### Consensus statements

- We recommend tailoring the care bundle for post-AKI/AKD outpatient follow-up according to the severity of both kidney and liver disease, with the delivery of care requiring close collaboration between hepatologists and nephrologists **(best practice statement).**
- We recommend personalised palliative care evaluation with goals including reduction in symptom burden, patient/caregiver wellbeing and goals of care discussions **(best practice statement).**

##### Rationale:

**Fig. 6.**
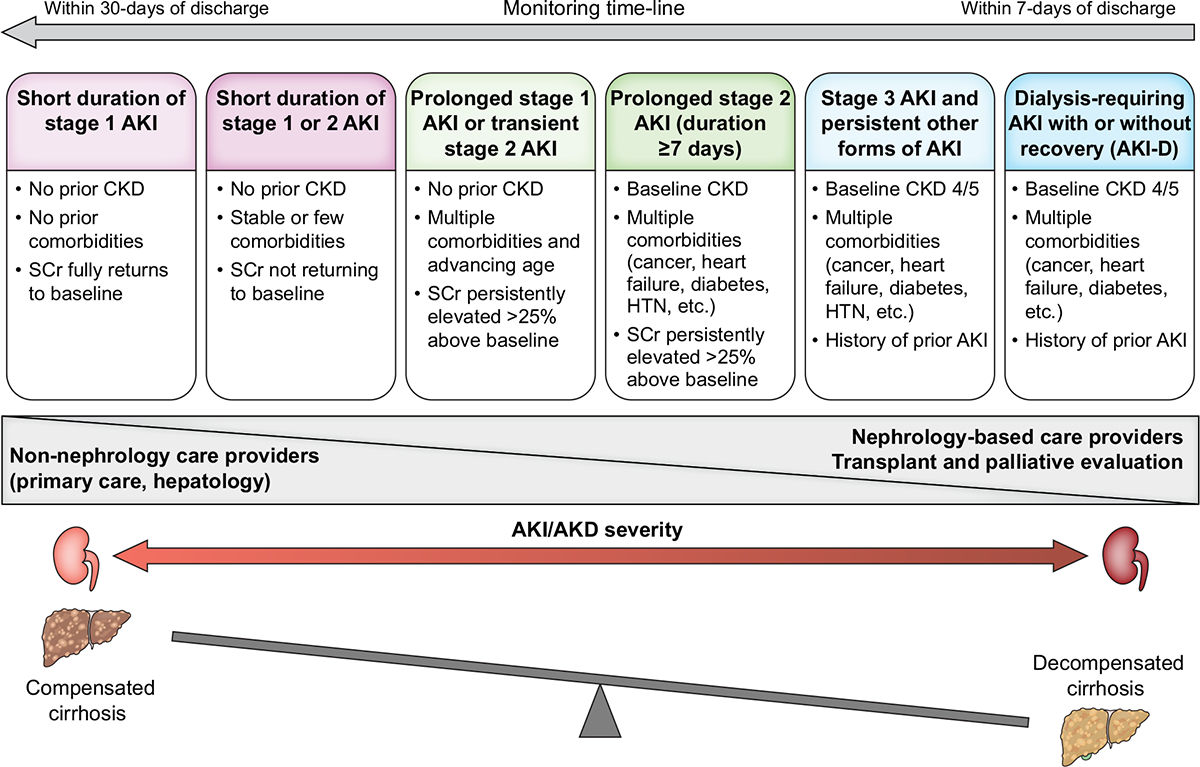
Recommended structure of post-discharge follow-up according to the evaluation of the kidney axis (severity, duration, and recovery of AKI) and the liver axis (compensated *vs*. decompensated cirrhosis) at the time of hospital discharge. Limited data are available to inform the timing and nature of monitoring for patients with cirrhosis who experience AKI or AKD in hospital. The post-discharge follow-up will depend on the state of kidney and liver health at the time of discharge. We suggest that these patients should have their kidney function checked within 1 month of hospital discharge, at a minimum, to confirm the extent of recovery or progression of kidney disease. Patients with persistent kidney dysfunction at 90 days should be formally assessed for the development or progression of CKD. Patients with less severe AKI or AKD can be monitored in primary care or by the base specialist with the degree of nephrology involvement in follow-up monitoring increasing with the duration and severity of AKI or AKD during hospitalization. Adapted, with permission, from Acute Disease Quality Initiative 24, www.ADQI.org. AKD, acute kidney disease; AKI, acute kidney injury; AKI-D, acute kidney injury treated with dialysis; CKD, chronic kidney disease; HTN, hypertension; NKD, no kidney disease; SCr, serum creatinine. Adapted from Acute Disease Quality Initiative 29, www.ADQI.org, CC BY 2.0 (https://creativecommons.org/licenses/by/2.0/)

Patients discharged following an episode of AKI are at an increased risk of recurrent episodes of AKI, progression to CKD, dialysis dependency and mortality.^197–202^ The post-hospital discharge period is a critical time in which dynamic liver and kidney function changes impact outcomes including mortality, transplant candidacy, and quality of life.^203^ Almost half of patients with cirrhosis discharged after an episode of AKI are re-admitted within 3 months, with 22% of readmissions due to renal and metabolic issues such as AKI, anasarca, or hyponatremia.^204^ Patients with cirrhosis who are discharged after an episode of AKI should have, at a minimum, a KLH assessment within 1 month of discharge, the timing of which can be individualised according to the risk-phenotype of the patient at the time of discharge (Fig. 6).^205^ From the clinical point of view, the stage of cirrhosis should be assessed during the first outpatient visit, with a focus on evaluating the presence and severity of specific complications. In assessing renal recovery, it is important to emphasise that using SCr may result in overestimation of kidney function and measurement of serum CysC levels should be considered when available.^206–208^ Screening for albuminuria should be routine following an episode of AKI as it has been shown to identify those patients who are at a higher risk of CKD progression.^209^

KLH management focuses on five key domains that include education, medication management, disease-modifying interventions, and dynamic transplant and palliative care evaluations. Communication of future risk of CKD and recurrent AKI episodes helps the patient adhere to preventative measures and avoid further kidney insults.^210^ Medication reconciliation is crucial during the first outpatient visit since dose modifications and initiation of medications such as diuretics are frequently necessary.^211^ The routine post-discharge evaluation of the global domains of frailty in addition to assessment of symptom burden and health-related quality of life, can allow for potentially modifiable gaps to be identified and addressed.^212,213^ More than one-third of caregivers of patients with cirrhosis have been shown to be affected by one or more major adverse impacts on their own lives and a substantial portion of family members are forced to stop working to provide care.^214^

Palliative care is not synonymous with end-of-life care and is associated with improvements in advance care planning, patient and caregiver satisfaction, and lower healthcare utilisation without detriment to patient survival, yet it remains underutilised in patients with cirrhosis (<5%), especially in patients never placed on the waiting list.^215–218^ Recognising AKI as a marker of worse prognosis in cirrhosis, persistent kidney dysfunction following discharge can be a trigger for reassessing goals of care by a multidisciplinary team, especially for those patients who are not candidates for LT and with a life expectancy of less than a few months.^215^ In such instances, a palliative approach to care is important to ensure that patient’s goals are elicited and translated into care that best meets their needs before an acute medical crisis occurs.

## Paediatric perspective

AKI is common in children with cirrhosis and carries significant morbidity and mortality.^219,220^ Cholestatic diseases such as biliary atresia are the leading aetiology of paediatric cirrhosis, precluding direct extrapolation of evidence from adult patients. The reported prevalence of paediatric HRS-AKI (<10%) is likely a gross underrepresentation as none of the proposed consensus definitions of HRS-AKI have been validated in children. CysC is a superior test for AKI detection and should be explored in combination with damage-related biomarkers to improve diagnostic accuracy in paediatric cirrhosis.^221^ Despite the different case-mix, the pathophysiology of HRS-AKI seems to be similar in children, thus paediatric HRS-AKI should be responsive to splanchnic vasoconstrictors like terlipressin.^222^ Paediatric cirrhotic cardiomyopathy, in part related to bile acids, contributes to the pathogenesis of AKI in cirrhosis and is associated with need for continuous RRT and post-transplant outcomes.^223,224^

## Conclusion and perspectives

AKI in patients with cirrhosis, especially HRS-AKI, is strongly associated with both short- and long-term adverse events. Over the last decade, there have been significant advances in our understanding of the pathophysiology and epidemiology of AKI in patients with cirrhosis. Our consensus recommendations are based not only on existing data but also on expert opinion, as much of the strength of evidence is poor and much evidence comes from studies in patients without cirrhosis. We acknowledge that some of the current literature contains limitations as many of the studies were performed prior to changes in the definition of HRS-AKI and further research is needed. However, utilising a multidisciplinary approach, we endeavoured to apply, as precisely as possible, lessons learned from AKI in the general population to the specific population of patients with cirrhosis. With the new diagnostic criteria for HRS-AKI, the integration (into routine practice) of appropriately selected biomarkers that can identify different sub-phenotypes of AKI should be increasingly explored, as this holds the key to further improvements in the care of patients with HRS-AKI. Consequently, it is imperative to develop research questions to address these knowledge gaps (Table S4). Overall, we believe that an integrated approach involving various specialties is imperative in the management of AKI in patients with cirrhosis, both in the inpatient and outpatient settings.

## Supplementary Material

Supplement

## Figures and Tables

**Table 1.** Strategies to prevent AKI in patients with cirrhosis.

Exposure	Preventive interventions
Iodinated contrast media exposure	Optimise fluid status to maintain euvolemia. There is no clear evidence to guide the optimal rate and duration of infusion of fluids. Hold diuretics only in patients with hypovolemia (diuretics may be continued in those with evidence of volume overload) No role for N-acetyl cysteine or urinary alkalinization with i.v. bicarbonate solutions Predisposing factors for CIN include female sex, presence of ascites, advanced liver disease, presence of infection and underlying kidney dysfunction^225–227^ Incidence of CIN is very low,^225–230^ and therefore i.v. contrast studies should not be withheld due to concerns regarding AKI where the information obtained could potentially have important therapeutic implications
Volume depletion (*e.g.*, diarrhoea, over diuresis)	Volume expansion with balanced solutions to correct hypovolemia Discontinue laxatives and/or diuretics
LVP (>5 L of ascites removed in a single session)	20–25% albumin solution (6–8 g for every litre over 5 L of ascites removed) to prevent post-paracentesis circulatory dysfunction
Variceal bleeding	Volume expansion with PRBCs if haemoglobin <8 g/dl^205^ Systematic antibiotics for 5–7 days Discontinue diuretics Consideration of pre-emptive TIPS in selected candidates^205^
Spontaneous bacterial peritonitis	Systematic administration of albumin with antibiotics Dose and duration of albumin administration should be titrated daily according to the patient’s volume and haemodynamic status to avoid under- and overresuscitation. Maintain MAP >60–65 mmHg in setting of septic shock
Bacterial infections other than spontaneous bacterial peritonitis	Volume expansion with crystalloid solutions, preferentially balanced solutions (*e.g.*, Lactated ringers, PlasmaLyte) in patients with sepsis-induced hypotension Caution with albumin administration to avoid volume overload and pulmonary oedema Dose of fluids should be administered according to the patient’s volume and haemodynamic status to avoid under- and over-resuscitation. Maintain MAP >60–65 mmHg in setting of septic shock
Nephrotoxic medications	Ensure kidney health by lessening the impact of drug-associated AKI events with prevention and optimal management including assessment of the nephrotoxic burden (*i.e.*, the sum of the number of nephrotoxins and the days of exposure to each) Ensure safe medication use with vigilant surveillance for drug-related events and avoid over- and under-dosing of drugs that are eliminated by the kidney (*i.e*., discontinuation, dose adjustment, alternative therapy) Correct dosing may be challenging as SCr concentration may not be representative of true kidney function. Recommend checking CysC (when available) for better estimation of kidney function
Major abdominal surgery	Monitor for postoperative ascites Optimise intravascular fluid status and avoid excessive sodium administration Avoid NSAIDs for pain control
alfapump^®^ (abdominal cavity to bladder pump for the treatment of ascites)	Caution with the initial daily volume removed by the pump that may be increased progressively (removal <1 L/day recommended) Not widely available in many countries

AKI, acute kidney injury; CIN, contrast-induced nephrotoxicity; LVP, large volume paracentesis; MAP, mean arterial pressure; NSAID, non-steroidal anti-inflammatory drug; PRBCs, packed red blood cells; SCr, serum creatinine.

**Table 2.** Vasoconstrictors used for the treatment of HRS-AKI.

Vasoconstrictor	Route/dose	Comments
Terlipressin	Continuous infusion*: 2–12 mg/day or i.v. bolus**: 1–2 mg every 6 h	Side effects include ischaemic events (cardiac, peripheral or mesenteric) and pulmonary oedema Cautious use in patients with evidence of intravascular volume overload
Norepinephrine	Continuous infusion: 0.5–3 mg/h	Consider as second-line agent if terlipressin contraindicated or unavailable Requires ICU care and central line placement Side effects include ischaemic events (cardiac, peripheral or mesenteric) and cardiac arrythmias.
Midodrine + octreotide	Oral: 7.5–15 mg every 8 h Subcutaneous: 100–200 μg every 8 h	Only consider if terlipressin is contraindicated or transfer to the ICU for norepinephrine infusion is not possible Midodrine may cause bradyarrhythmias
**Criteria for discontinuation** SCr within 0.3 mg/dl of baseline No improvement in SCr after 48–72 h with maximal tolerated doses Serious adverse reaction Initiation of RRT Liver transplantation Total duration of 14 days		
**Dose titration** Terlipressin dose should be increased by at least 2 mg/day every 24 h for those on continuous infusion up to a maximum of 12 mg/day and increased from 1 to 2 mg every 6 h for i.v. bolus, if SCr has not improved by 25%. Norepinephrine dose is increased every 4 h in steps of 0.5 mg/h, up to the maximum dose of 3 mg/h if no increase in MAP >10 mmHg or UO 50 ml/h × 4 h Daily 20–25% albumin (20–40 g/day) is recommended, however, amount and dose should be adjusted daily based on patients’ volume status. Albumin should be withheld if evidence of fluid overload and/or pulmonary oedema		

All vasoconstrictors are given in combination with albumin.

ICU, intensive care unit; MAP, mean arterial pressure; RRT, renal replacement therapy; SCr, serum creatinine; UO, urine output.

* Continuous infusion of terlipressin may be associated with a lower incidence of side effects compared to i.v. bolus, most likely due to lower cumulative daily dose.^178^.

** 1 vial = 0.85 mg terlipressin (North American FDA label) = 1 mg terlipressin acetate.

## Key points:

- According to the Acute Disease Quality Initiative (ADQI) and International Club of Ascites (ICA) joint multidisciplinary consensus meeting, acute kidney injury (AKI) in patients with cirrhosis is defined using KDIGO criteria: increase in serum creatinine ≥0.3 mg/dl (26.5 μmol/L) within 48 h or ≥50% from baseline value known or presumed to have occurred within the prior 7 days and/or urine output ≤0.5 ml/kg for ≥6 h.
- The lowest, stable serum creatinine value obtained in the previous 3 months may be used for the diagnosis and staging of AKI. If no values are available in the previous 3 months, the most recent value up to 12 months prior may be used.
- Hepatorenal syndrome-AKI (HRS-AKI) is a phenotype of AKI that is specific to patients with advanced cirrhosis and ascites, which may also occur in the presence of tubular injury, proteinuria, and/or pre-existing chronic kidney disease.
- The following diagnostic criteria for HRS-AKI should be: a) cirrhosis with ascites; b) increase in serum creatinine ≥0.3 mg/dl (26.5 μmol/L) within 48 h or ≥50% from baseline value, known or presumed, to have occurred within the prior 7 days and/or urine output ≤0.5 ml/kg for ≥6 h; c) absence of improvement in serum creatinine and/or urine output within 24 h following adequate volume resuscitation (when clinically indicated); and d) absence of strong evidence for an alternative explanation as the primary cause of AKI.
- The ADQI and ICA joint multidisciplinary consensus meeting recommends against systematic administration of albumin for 48 h as a requisite for the diagnosis of HRS-AKI.
- Vasoconstrictor therapy (terlipressin as first-line agent), in combination with 20–25% albumin, should be initiated immediately upon establishing a diagnosis of HRS-AKI.

## Box 1. ICA-ADQI new diagnostic criteria for HRS-AKI.

- Cirrhosis with ascites
- Increase in serum creatinine ≥0.3 mg/dl (26.5 μmol/L) within 48 h or ≥50% from baseline value known or presumed to have occurred within the prior 7 days and/or urinary output ≤0.5 ml/kg for ≥6 h
- Absence of improvement in serum creatinine and/or urine output within 24 h following adequate volume resuscitation (when clinically indicated)
- Absence of strong evidence for an alternative explanation as the primary cause of AKI

Presence of underlying kidney disease does not exclude a diagnosis of superimposed HRS-AKI and HRS-AKI may coexist with other causes of AKI. Examples of alternative causes of AKI include septic shock requiring vasopressors, drug-induced AKI, obstruction, or acute glomerular injury. Patients who meet HRS criteria are considered to have HRS-AKI, HRS-AKD or HRS-CKD based on timing and duration of kidney dysfunction. ADQI, Acute Disease Quality Initiative; AKD, acute kidney disease; AKI, acute kidney injury; CKD, chronic kidney disease; HRS, hepatorenal syndrome; ICA, International Club of Ascites.

## Abbreviations

ACLF: acute-on-chronic liver failure
ADQI: Acute Disease Quality Initiative
AKI: acute kidney injury
AKD: acute kidney disease
ATI: acute tubular injury
ATN: acute tubular necrosis
CKD: chronic kidney disease
CKD-EPI: chronic kidney disease epidemiology collaboration
CO: cardiac output
CysC: cystatin C
FENa: fractional excretion of sodium
GFR: glomerular filtration rate
HRS: hepatorenal syndrome
ICA: International Club of Ascites
ICU: intensive care unit
KDIGO: Kidney Disease: Improving Global Outcomes
KLH: kidney-liver health
LT: liver transplantation
LVP: large volume paracentesis
MAP: mean arterial pressure
MELD: model for end-stage liver disease
PAMP: pathogen-associated molecular pattern
RCT: randomised-controlled trial
RRT: renal replacement therapy
SBP: spontaneous bacterial peritonitis
SCr: serum creatinine
SLK: simultaneous liver and kidney
TIPS: transjugular intrahepatic portosystemic shunts
uNGAL: urinary neutrophil gelatinase-associated lipocalin
UO: urine output
