# Supplementary material for: Acute kidney injury in patients with cirrhosis: Acute Disease Quality Initiative (ADQI) and International Club of Ascites (ICA) joint multidisciplinary consensus meeting
Source: J Hepatol. Author manuscript; Available in PMC 2024 Jul 1. (PMC11193657; doi:10.1016/j.jhep.2024.03.031)
Supplement: Supplement [file NIHMS1991444-supplement-Supplement.pdf]

Journal of Hepatology, Volume ■

Supplemental information

Acute kidney injury in patients with cirrhosis: Acute Disease Quality Initiative (ADQI) and International Club of Ascites (ICA) joint multidisciplinary consensus meeting

Mitra K. Nadim, John A. Kellum, Lui Forni, Claire Francoz, Sumeet K. Asrani, Marlies Ostermann, Andrew S. Allegretti, Javier A. Neyra, Jody C. Olson, Salvatore Piano, Lisa B. VanWagner, Elizabeth C. Verna, Ayse Akcan-Arikan, Paolo Angeli, Justin M. Belcher, Scott W. Biggins, Akash Deep, Guadalupe Garcia-Tsao, Yuri S. Genyk, Pere Gines, Patrick S. Kamath, Sandra L. Kane-Gill, Manish Kaushik, Nuttha Lumlertgul, Etienne Macedo, Rakhi Maiwall, Sebastian Marciano, Raimund H. Pichler, Claudio Ronco, Puneeta Tandon, Juan-Carlos Q. Velez, Ravindra L. Mehta, and François Durand

Acute kidney injury in patients with cirrhosis: Acute Disease Quality Initiative (ADQI) and International Club of Ascites (ICA) joint multidisciplinary consensus meeting☆

Mitra K. Nadim, John A. Kellum, Lui Forni, Claire Francoz, Sumeet K Asrani, Marlies Ostermann, Andrew S. Allegretti, Javier A. Neyra, Jody C. Olson, Salvatore Piano, Lisa B. VanWagner, Elizabeth C. Verna, Ayse Akcan-Arikan, Paolo Angeli, Justin M. Belcher, Scott W. Biggins, Akash Deep, Guadalupe Garcia-Tsao, Yuri S. Genyk, Pere Gines, Patrick S. Kamath, Sandra L. Kane-Gill, Manish Kaushik, Nuttha Lumlertgul, Etienne Macedo, Rakhi Maiwall, Sebastian Marciano, Raimund H. Pichler, Claudio Ronco, Puneeta Tandon, Juan-Carlos Q. Velez, Ravindra L. Mehta, François Durand

Table of contents

Table S1……………………………………………………………………………………2

Table S2……………………………………………………………………………………3

Table S3……………………………………………………………………………………5

Table S4……………………………………………………………………………………9

Supplementary references………………………………………………………………11

Table S1: GRADE system for grading recommendations according to strength of recommendation and quality of evidence.[1-3]

**Strength of Recommendation**		
**Strong**<br>**'We recommend'**	Conditions for which there is evidence and/or general agreement that a given procedure or treatment is beneficial, useful and effective	
**Weak**<br>**'We suggest'**	Conditions for which there is conflicting evidence and/or divergence of opinion about the usefulness/efficacy of a procedure or treatment	
**Quality of Evidence and Definitions**		**Symbol**
**High**	Further research is very unlikely to change our confidence in the estimate effect	**A**
**Moderate**	Further research is likely to have an important impact on our confidence in the estimate of effect and may change the estimate	**B**
**Low**	Further research is very likely to have an important impact on our confidence in the estimate of effect and is likely to change the estimate	**C**
**Very low**	Any estimate of effect is very uncertain	**D**
**Best Practice Statement**	Actionable statements deemed to be necessary for practice (desirable effects of an intervention clearly outweigh its undesirable effects) but are supported by indirect evidence that does not diminish the certainty in evidence.	

Table S2: Clinical utility and limitation of AKI biomarkers in patients with cirrhosis

Biomarker	Clinical utility	Limitations
**AKI Stress Markers**		
TIMP-2 x IGFBP-7 (urine)	FDA-approved and CE-marked test to predict the risk of developing AKI stage 2/3 within 12 hours of assessment	Small studies in patients with decompensated cirrhosis have not shown the test to predict early AKI[4] or HRS-AKI[5]; Urine albumin >125 mg/dL interferes with the result and >3000 mg/dL invalidates it; Bilirubinuria (conjugated) at concentrations exceeding 72mg/L interferes with the result[6]
**AKI Functional Biomarkers**		
Creatinine (SCr) (serum)	Most commonly used biomarker of kidney function and assay available in all clinical laboratories; AKI is currently defined and staged according to the changes in SCr; Used in MELD score to prioritize candidates for liver transplantation	Overestimates GFR in patients with cirrhosis because of decreased creatinine production, protein calorie malnutrition, and muscle wasting; Overestimates GFR in patients who are fluid overloaded, including presence of ascites which increases the total volume of distribution
Cystatin C (plasma)	Rise often precedes increase in SCr and demonstrates better diagnostic accuracy than SCr in patients with cirrhosis; Not impacted by liver function, muscle mass, bilirubin or age; Combined MELD CysC score shown to improve prognostic accuracy of MELD alone for 1-year mortality[7]; eGFR formulas that incorporate CysC and Scr perform better compared to Scr based formulas alone	May be affected by active inflammation and steroid use; Not widely available
Proenkephalin (PenKid) (serum)	CE-marked for clinical evaluation of glomerular function; Increased plasma concentrations strongly correlate with reduced GF[8] and are unaffected by systemic critical illness, inflammation, age or gender ; Detects AKI 48 hours prior to rise in Scr in patients undergoing liver transplantation and predictor of severe AKI [9]	Lacking validation studies in patients with cirrhosis
**AKI Damage Markers**		
Albuminuria / Proteinuria	Dipstick used as screening tool for diagnosis of kidney disease due to low cost, wide availability, and ability to provide quick point-of-care information; Albuminuria (urine albumin to creatinine ratio) used to identify risk for CKD; Has been shown to discriminate HRS-AKI from ATI (> 44 mg/dL, AUC 0.73-0.86)[10] and predict 'subclinical AKI' (> 80 mg/g creatinine)	Studies in discriminating HRS-AKI from ATI and prediction of subclinical AKI are small and exact cut-off needs further investigation. Interpretation complicated by pre-existing proteinuria
NGAL (urine or serum)	uNGAL measured day 3 (cutoff 220-244 µg/g creatinine, Bioporto assay) shows greatest accuracy for distinguishing ATI from HRS-AKI;[11]; Elevated uNGAL associated with AKI progression in patients with cirrhosis; Addition of uNGAL to	Lack of availability in many countries; Kinetic profile and clinical utility depend on type of NGAL measured and patient population studied; Lack of specific cut-off

	MELD may predict mortality more accurately compared to MELD alone, in patients with decompensated cirrhosis; May predict AKI following paracentesis;[12] uNGAL pick-up 'subclinical AKI" in patients at risk for AKI[4,13-15]	values; Cut-off values vary depending on assay used; Overlap with ATN and HRS-AKI; Levels in urine increase with urinary tract infection
KIM-1 (urine or serum)	Released into urine 12-24 hours after tubular injury with peak at 2-3 days; Expression significantly upregulated in kidney after ischemia-reperfusion injury and drug-induced AKI; FDA-approved as an AKI biomarker for drug development; Serum levels significantly higher in patients with ATN compared to HRS-AKI;[10,16] Predictor of mortality in patients with cirrhosis and AKI	Lack of specific cut-off values; Overlap with ATN and HRS-AKI
IL-18 (urine or serum)	Pro-inflammatory cytokine released into urine after tubular damage Role in diagnosis and prognostication of AKI, and differentiating ATI from HRS[10,16,17]	Lack of specific cut-off values; Overlap with ATI and HRS-AKI Outperformed by uNGAL in a prospective study;[11] Increased with inflammation
L-FABP (urine)	Role in diagnosis and prognostication of AKI, and differentiating ATN from HRS[10,16,17] and development of AKI and ACLF in patients with decompensated cirrhosis[18]	Not specific to kidney injury; Lack of specific cut-off values; Overlap with ATI and HRS-AKI; Increased in patients with CKD and sepsis
**Other AKI Markers**		
FeNa / FeUrea (urine)	Test used to differentiate between pre-renal AKI, ATI and HRS-AKI FENa <0.2%[10] and FEUrea < 21%[19] has been shown to discriminate HRS-AKI from ATI	Most laboratories do not report urinary sodium < 20 mEq/L; FENa influenced by diuretic use and underlying CKD; Overlap with ATI and HRS-AKI
Urine microscopy	Commonly used tool to diagnose specific etiologies of AKI	Prone to inter-observer variability;[20] Normal urine microscopy does not rule out presence of significant parenchymal injury in patients with cirrhosis;[21] Granular casts does not rule out HRS-AKI
Renal biopsy	Determining etiology of AKI and degree of chronic damage	Bleeding risk in patients with coagulopathy and thrombocytopenia (less likely via transjugular)

This table is not intended to be an exhaustive list of biomarkers[22,23] but rather a compilation of currently available and more commonly studied biomarkers in AKI in patients with cirrhosis. AKI, acute kidney injury; ACLF, acute on chronic liver failure; ATI, acute tubular injury; CCL14, C–C chemokine ligand 14; CE, Conformité Européenne (European Conformity); CKD, chronic kidney disease; CysC, cystatin C; FDA, food and drug administration; FENa, fractional excretion of sodium; FEUrea, fractional excretion of urea; GFR, glomerular filtration rate; HRS, hepatorenal syndrome; IGFBP7, insulin-like growth factor binding protein; KIM-1, kidney injury molecule 1; MELD, model of end-stage liver disease; NGAL, neutrophil gelatinase-associated lipocalin; penKID, proenkephalin A 119-159; SCr, serum creatinine; TIMP-2, tissue inhibitor of metalloproteinase-2

Table S3: Use of albumin in patients with decompensated cirrhosis: a comparison of randomized control trials

Study (Year)	Country / # of Patients	Treatment Arms (Duration)	Results
Large Volume Paracentesis			
Gines [24] (1988)	Spain (Single center) N=105	– Paracentesis (4–6 L/day) + 20% Alb (40 g each tap) vs paracentesis alone	– Significant increase in BUN and development of hyponatremia in the group not receiving Alb.
Planas et al.[25] (1990)	Spain (4 centers) N=88	– 20% Alb vs Dextran-70, both given at a dose of 8 g/L of ascites removed	– 18% with SCr >1.5 mg/dL<br>– No difference in incidence of renal impairment, hyponatremia, HE, GIB, bacterial infections, hospital readmission, or mortality
Salerno et al.[26] (1991)	Italy (Single center) N=54	– Haemaccel (polygeline 3.5%) (150 ml/L of ascites evacuated) vs 20% Alb (6 g/L of ascites removed)<br>–	– 22% with renal impairment at baseline (defined as 50% increase in SCr, to a level higher than 1.2 mg/dl)<br>– No difference in incidence of renal impairment, hyponatremia, HE, GIB, survival, re-hospitalization for recurrent massive ascites
Fassio et al.[27] (1992)	Argentina (2 centers) N=44	– Dextran-70 vs 20% Alb; 6 gm/L ascites (Daily paracentesis up to 5 L of ascitic fluid)	– No difference in kidney and liver function tests 96 hrs after paracentesis<br>– No difference in re-admission or mortality
Gines et al.[28] (1996)	Spain (12 centers) N=289	– Dextran-70 vs. Polygeline vs 20% Alb; all given at 8 gm/L of ascites fluid removed	– 17% with SCr > 1.5 mg/dL<br>– PPCD rates significantly higher in patients treated with Dextran and polygeline group compared to Alb group when > 5L of ascitic fluid removed.<br>– SCr significantly higher and serum sodium significantly lower in Dextran and Polygeline groups compared to Alb
Altman et al.[29] (1998)	France (5 centers) N=65	– HES (32.5 gm if < 2 L and 65 gm if 2-5 L of ascites removed)	– No difference in development of kidney dysfunction or hyponatremia

		vs 20% Alb (20 gm if < 2 L and 40 gm if 2-5 L ascites removed).<br>- Paracentesis repeated daily (up to 5L/day)	
Garcia-Compean et al.[30] (2002)	France (2 centers) N=69	- Dextran-40 vs 20% Alb, both given at 8 gm/L ascites removed.	- 28% with renal impairment at baseline.<br>- Significantly more PPCD with Dextran compared to Alb<br>- No difference in rates of renal impairment, hyponatremia, survival or recurrence of ascites between the two groups
Sola-Vera et al.[31] (2003)	Spain (2 centers) N=72	- 3.5% saline solution vs 20% Alb<br>- Cross-over study: patients admitted with a second episode of tense ascites received alternative plasma expander	- 9.7% with SCr > 1.5 mg/dL at baseline.<br>- Higher incidence of PPCD in saline group when ≥ 6 L ascites removed<br>- No difference in incidence of renal impairment or hyponatremia.
Moreau et al.[32] (2006)	France (18 centers) N=68	- 3.5% Polygeline vs 20% albumin	- Lower rates (not statistically significant) of renal impairment and hyponatremia in Alb group<br>- Trial prematurely discontinued because of safety concerns about bovine-derived products that emerged during the study period
Spontaneous Bacterial Peritonitis			
Sort et al.[33] (1999)	Spain (7 centers) N=199	- Abx + 20% Alb (1.5 g/kg day 1 then 1 g/kg day 3) vs Abx alone	- 40% with SCr > 1.5 mg/dL at baseline<br>- No difference in SBP resolution<br>- Renal impairment, hospital and 3-month mortality lower in Alb group<br>- Incidence of renal impairment in patients with serum bilirubin <4 mg/dL, SCr <1 mg/dL, and BUN < 30 mg/dL very low
Chen et al.[34] (2009)	Taiwan (Single center) N=30	- Abx + 20% albumin (50 ml on day 1-3) vs Abx alone	- Trend towards lower rates of renal impairment and hospital mortality in Alb group (did not reach statistical significance)
Non-Spontaneous Bacterial Peritonitis Infections			

Guevara et al.[35] (2012)	Spain (Single center) N=102	- Abx + 20% Alb (1.5 gm/kg on day 1, 1 gm/kg on day 3) vs Abx alone	- 21% with renal impairment at baseline.<br>- No difference in 3-month mortality, renal impairment, or recovery<br>- Risk of pulmonary edema 5.3% in Alb group
Thévenot et al.[36] (2015)	France (25 centers) N=191	- Abx + 20% albumin (1.5 gm/kg on Day 1 followed by 1 gm/kg on Day 3) vs Abx alone	- No difference in 3-month mortality or renal impairment<br>- Renal impairment lower in patients with severe sepsis receiving Alb<br>- Pulmonary edema: 8.3% in the Alb group, 2 of whom died.
Fernandez et al.[37] (2020)	EASL-CLIF Consortium (27 centers) N=118	- Abx + 20% Alb vs Abx alone<br>- Alb dose : 1.5 g/kg Day 1, 1 g/kg Day 3	- 76% with SCr >=1.2 mg/dl<br>- No difference in hospital or 90-day mortality, resolution of infection, resolution of kidney dysfunction<br>- Higher rate of ACLF resolution in Alb group<br>- Pulmonary edema more frequent in Alb group (P=0.12)
Critically Ill Patients with Hypoalbuminemia (< 3.0 g/dL)			
China et al.[38] (ATTIRE) (2021)	United Kingdom (35 centers) N=777	- 20% Alb at 100 mL/hour to maintain serum albumin >3.5 g/dL<br>- Alb Protocol: 100 ml if Alb <3.4 g/dL; 200 ml if Alb <2.9 g/dL; 300 ml if f Alb <2.5 g/dL; 400 ml if Alb <2.0 g/dL	- 10.5% with SCr ≥1.5 mg/dl<br>- Alb group received 200 g (IQR 140-280) vs. control 20 g (IQR 0-120)<br>- No difference in kidney dysfunction (defined as KDIGO AKI SCr criteria or initiation of RRT), incidence of new infections, or mortality (28-day, 3-month or 6-month)<br>- Risk of pulmonary edema and fluid overload greater in Alb group
Critically ill patients with Sepsis-induced Hypotension			
Philips et al.[39] (FRISC) (2021)	India (Single center) N=308	- 250 mL of 5% Alb followed by maintenance infusion at 50 mL/hr for 3 hrs vs 0.9% NaCl 30 mL/kg followed by maintenance infusion at 100 mL/hr for 3 hrs	- 73% with AKI at baseline (Scr > 1.5 mg/dL)<br>- At 3 hours:<br>Improvement in hemodynamic parameters, lactate dynamics higher in Alb group but did not confer long-lasting benefits<br>- No difference in UO<br>- 1-week survival higher in the Alb group compared to saline
Maiwall et al.[40] (ALPS) 2022	India (Single center) N=100	- 20% Alb 0.5-1.0 gm/kg over first 3 hrs versus 30 mL/kg Plasmalyte-148 over 3 hours	- Baseline Scr (mg/dL) 2.08 ± 0.93 Alb group and 2.49 ± 1.51 plaslmalyte group<br>- Quicker improvement in hemodynamics and lactate clearance with Alb<br>- No difference in 28- day mortality, SCr or need for RRT at 7 days

			– Higher rate of pulmonary complication in Alb group<br>– Alb discontinued in 22% patients due to volume overload<br>– Significantly lower cumulative fluids administered at 24 hours in the Alb group 616.50 ± 586.64 ml vs. 3710 ± 1550 mL in the plasmalyte group (p <0.001).
Long term use of Albumin in Patients with Decompensated Cirrhosis			
Caraceni et al.[41] (ANSWER) (2018)	Italy (33 sites) N=431	– SMT + 20% Alb (40 g) 2x/week for 2 weeks, then 40 g weekly vs SMT in patients with uncomplicated ascites<br>– Duration of treatment: 18 months	– 6% with SCr > 1.5 mg/dL at baseline.<br>– Lower incidence of SBP, non-SBP bacterial infections, AKI / HRS-AKI, HE grade 3 or 4, refractory ascites and hyponatraemia and mortality in Alb group<br>– Alb group seen more frequently than SMT group, and thus, impending complications could have been treated more promptly<br>– Alb associated with better quality of life and fewer hospital admissions<br>– Pulmonary edema 3% Alb vs 1% control group (p=0.163)
Solà et al.[42] (MACHT) (2018)	Spain (3 sites) N=173	– Midodrine (15-30 mg/day) + 20% Alb (40 gm) every 15 days vs midodrine + placebo (tablets + 0.9% saline) in patients with uncomplicated ascites<br>– Duration: 12 months	– Renal impairment: 13% at baseline.<br>– Median duration of treatment 80 days due to transplantation<br>– No difference in complications of cirrhosis or 1-year mortality<br>– Only 10% in Alb group and 23% in control completed study period of 12 months, p=0.023)<br>– No difference in Alb level at end of study

Abbreviations: ACLF, acute on chronic liver failure; AKI, acute kidney injury; Alb, albumin; EASL-CLIF, European Association for the Study of the Liver-Chronic Liver Failure; GIB, gastrointestinal bleed; HE, hepatic encephalopathy; HES, hydroxyethyl starch; HRS, hepatorenal syndrome; ICU, intensive care unit; LVP, large volume paracentesis; MAP, mean arterial pressure; PPCD, post-paracentesis circulatory dysfunction; SBP, spontaneous bacterial peritonitis; SCr, serum creatinine; SMT, standard medical therapy; UO, urine output

Table S4. Knowledge gaps and future research directions in patients with cirrhosis and kidney disease

Knowledge Gap	Future Research Directions
**Epidemiology & Definitions**	1. What is the epidemiology, risk factors, and renal and non-renal outcomes of patients based on the new diagnostic criteria for HRS-AKI?<br>2. How should UO be evaluated in patients with cirrhosis and AKI (i.e. use of actual or ideal body weight, strict time period or time-averaged values?<br>3. Determine the best method for determining reference SCr, especially in the absence of any previous SCr value
**Pathophysiology**	1. Determination of an identifiable timepoint during which impaired cardiac contractile response "gives way" to decline in cardiac output that can guide therapeutic interventions.<br>2. What are the mechanisms of adaptive and maladaptive repair in AKI in patients with cirrhosis and how do they influence the trajectory of renal recovery?<br>3. What are the pathophysiological mechanisms that determine response to treatment and renal recovery in AKI, in particular HRS-AKI, in patients with decompensated cirrhosis?
**Prevention and workup of AKI**	1. Determine the optimal dose and duration of treatment with albumin necessary for the prevention of AKI in patients with SBP or LVP.<br>2. Determine patient population that would benefit from long-term outpatient albumin administration<br>3. Determine the specific cut-off's for uNGAL predictive of response to treatment of HRS-AKI.<br>4. Specific estimated glomerular filtration rate equations need to be developed in patients with cirrhosis.<br>5. Prospective study on the role of cystatin-C compared to SCr in timely diagnosis of AKI and predicting waitlist mortality.
**Treatment of AKI**	1. Determine the optimal dose, concentration, duration, and stopping criteria for the use of intravenous albumin in patients with HRS-AKI<br>2. Define starting and stopping criteria for volume resuscitation in patients with AKI using real-time and objective measures of intravascular volume, such as point of care ultrasound.<br>3. Prospective studies to determine optimal timing for vasoconstrictor initiation, titration and discontinuation, based on SCr, MAP, or other biomarkers.

	4. Further prospective studies comparing safety, efficacy and cost of continuous versus bolus terlipressin dosing<br>5. Prospective controlled studies to determine the MAP rise target with the most optimal balance of safety and efficacy.<br>6. Studies using extracorporeal liver support for the management of AKI as a bridge to liver transplantation or recovery of synthetic liver function.
**Post-discharge AKI Follow-up**	1. Investigate the natural history of patients with cirrhosis that survived an episode of hospitalized AKI and determine the relevant risk factors and clinical and patient-centered outcomes that should be targeted in interventional studies<br>2. Prospective and interventional studies to address care processes and outcomes in order to improve the overall health and well-being of patients with cirrhosis after an episode of AKI.<br>3. Frameworks of eliciting patients' goals of care and provider instruction on serious illness conversations in this patient population are needed.
**Pediatric Population**	1. Validation of proposed definitions of HRS-AKI and HRS-AKD in pediatric populations and prognostic and predictive enrichment with combined damage and functional biomarkers are needed for timely detection of AKI phenotypes, assessment of vasoconstrictor responsiveness and optimal dose and duration of therapy.<br>2. Renal and global outcomes of AKI survivors need longitudinal study to assess life-course impact, including later development of CKD post-transplant and overall functional status and quality of life.

AKI, acute kidney injury; CKD, chronic kidney disease; HRS, hepatorenal syndrome; LVP, large volume paracentesis; MAP, mean arterial pressure; SCr, serum creatinine; SBP, spontaneous bacterial peritonitis; uNGAL, urine neutrophil gelatinase-associated lipocalin

Supplementary references

1. Guyatt GH, Oxman AD, Kunz R, et al. Going from evidence to recommendations. BMJ 2008;336(7652):1049-51. DOI: 10.1136/bmj.39493.646875.AE.
2. Dewidar O, Lotfi T, Langendam MW, et al. Good or best practice statements: proposal for the operationalisation and implementation of GRADE guidance. BMJ Evid Based Med 2023;28(3):189-196. DOI: 10.1136/bmjebm-2022-111962.
3. Guyatt GH, Oxman AD, Vist GE, et al. GRADE: an emerging consensus on rating quality of evidence and strength of recommendations. BMJ 2008;336(7650):924-6. DOI: 10.1136/bmj.39489.470347.AD.
4. Jo SK, Yang J, Hwang SM, Lee MS, Park SH. Role of biomarkers as predictors of acute kidney injury and mortality in decompensated cirrhosis. Sci Rep 2019;9(1):14508. DOI: 10.1038/s41598-019-51053-8.
5. Zhang CC, Hoffelt DAA, Merle U. Urinary cell cycle arrest biomarker [TIMP-2].[IGFBP7] in patients with hepatorenal syndrome. Biomarkers 2019;24(7):692-699. DOI: 10.1080/1354750X.2019.1652347.
6. Uettwiller-Geiger DL, Vijayendran R, Kellum JA, Fitzgerald RL. Analytical characteristics of a biomarker-based risk assessment test for acute kidney injury (AKI). Clin Chim Acta 2016;455:93-8. DOI: 10.1016/j.cca.2016.01.012.
7. Maiwall R, Kumar A, Bhardwaj A, Kumar G, Bhadoria AS, Sarin SK. Cystatin C predicts acute kidney injury and mortality in cirrhotics: A prospective cohort study. Liver Int 2018;38(4):654-664. DOI: 10.1111/liv.13600.

8. Khorashadi M, Beunders R, Pickkers P, Legrand M. Proenkephalin: A New Biomarker for Glomerular Filtration Rate and Acute Kidney Injury. Nephron 2020;144(12):655-661. DOI: 10.1159/000509352.

9. Lima C, Gorab DL, Fernandes CR, Macedo E. Role of proenkephalin in the diagnosis of severe and subclinical acute kidney injury during the perioperative period of liver transplantation. Pract Lab Med 2022;31:e00278. DOI: 10.1016/j.plabm.2022.e00278.

10. Belcher JM, Sanyal AJ, Peixoto AJ, et al. Kidney biomarkers and differential diagnosis of patients with cirrhosis and acute kidney injury. Hepatology 2014;60(2):622-32. (In eng). DOI: 10.1002/hep.26980.

11. Huelin P, Sola E, Elia C, et al. Neutrophil Gelatinase-Associated Lipocalin for Assessment of Acute Kidney Injury in Cirrhosis: A Prospective Study. Hepatology 2019;70(1):319-333. DOI: 10.1002/hep.30592.

12. Pietrukaniec M, Migacz M, Zak-Golab A, et al. Could KIM-1 and NGAL levels predict acute kidney injury after paracentesis? - preliminary study. Ren Fail 2020;42(1):853-859. DOI: 10.1080/0886022X.2020.1801468.

13. Slack AJ, McPhail MJ, Ostermann M, et al. Predicting the development of acute kidney injury in liver cirrhosis--an analysis of glomerular filtration rate, proteinuria and kidney injury biomarkers. Aliment Pharmacol Ther 2013;37(10):989-97. DOI: 10.1111/apt.12299.

14. Treeprasertsuk S, Wongkarnjana A, Jaruvongvanich V, et al. Urine neutrophil gelatinase-associated lipocalin: a diagnostic and prognostic marker for acute kidney injury (AKI) in hospitalized cirrhotic patients with AKI-prone conditions. BMC Gastroenterol 2015;15:140. DOI: 10.1186/s12876-015-0372-5.

15. Cho E, Kim SC, Kim MG, Jo SK, Cho WY, Kim HK. The incidence and risk factors of acute kidney injury after hepatobiliary surgery: a prospective observational study. BMC Nephrol 2014;15:169. DOI: 10.1186/1471-2369-15-169.

16. Belcher JM, Garcia-Tsao G, Sanyal AJ, et al. Urinary biomarkers and progression of AKI in patients with cirrhosis. Clin J Am Soc Nephrol 2014;9(11):1857-67. DOI: 10.2215/CJN.09430913.

17. Puthumana J, Ariza X, Belcher JM, Graupera I, Gines P, Parikh CR. Urine Interleukin 18 and Lipocalin 2 Are Biomarkers of Acute Tubular Necrosis in Patients With Cirrhosis: A Systematic Review and Meta-analysis. Clin Gastroenterol Hepatol 2017;15(7):1003-1013 e3. DOI: 10.1016/j.cgh.2016.11.035.

18. Juanola A, Graupera I, Elia C, et al. Urinary L-FABP is a promising prognostic biomarker of ACLF and mortality in patients with decompensated cirrhosis. J Hepatol 2022;76(1):107-114. DOI: 10.1016/j.jhep.2021.08.031.

19. Patidar KR, Kang L, Bajaj JS, Carl D, Sanyal AJ. Fractional excretion of urea: A simple tool for the differential diagnosis of acute kidney injury in cirrhosis. Hepatology 2018;68(1):224-233. DOI: 10.1002/hep.29772.

20. Palsson R, Colona MR, Hoenig MP, et al. Assessment of Interobserver Reliability of Nephrologist Examination of Urine Sediment. JAMA Netw Open 2020;3(8):e2013959. DOI: 10.1001/jamanetworkopen.2020.13959.

21. Trawale JM, Paradis V, Rautou PE, et al. The spectrum of renal lesions in patients with cirrhosis: a clinicopathological study. Liver Int 2010;30(5):725-32. DOI: 10.1111/j.1478-3231.2009.02182.x.

22. Asrani SK, Shankar N, da Graca B, Nadim MK, Cardenas A. Role of Novel Kidney Biomarkers in Patients With Cirrhosis and After Liver Transplantation. Liver Transpl 2022;28(3):466-482. DOI: 10.1002/lt.26344.

23. Francoz C, Nadim MK, Durand F. Kidney biomarkers in cirrhosis. J Hepatol 2016;65(4):809-824. (In eng). DOI: 10.1016/j.jhep.2016.05.025.

24. Ginès P, Titó L, Arroyo V, et al. Randomized comparative study of therapeutic paracentesis with and without intravenous albumin in cirrhosis. Gastroenterology 1988;94(6):1493-502. (In eng). DOI: 10.1016/0016-5085(88)90691-9.

25. Planas R, Gines P, Arroyo V, et al. Dextran-70 versus albumin as plasma expanders in cirrhotic patients with tense ascites treated with total paracentesis. Results of a randomized study. Gastroenterology 1990;99(6):1736-44. DOI: 10.1016/0016-5085(90)90481-f.

26. Salerno F, Badalamenti S, Lorenzano E, Moser P, Incerti P. Randomized comparative study of hemaccel vs. albumin infusion after total paracentesis in cirrhotic patients with refractory ascites. Hepatology 1991;13(4):707-13. (In eng).

27. Fassio E, Terg R, Landeira G, et al. Paracentesis with Dextran 70 vs. paracentesis with albumin in cirrhosis with tense ascites. Results of a randomized study. J Hepatol 1992;14(2-3):310-6. (In eng). DOI: 10.1016/0168-8278(92)90176-p.

28. Gines A, Fernandez-Esparrach G, Monescillo A, et al. Randomized trial comparing albumin, dextran 70, and polygeline in cirrhotic patients with ascites treated by paracentesis. Gastroenterology 1996;111(4):1002-10. DOI: 10.1016/s0016-5085(96)70068-9.

29. Altman C, Bernard B, Roulot D, Vitte RL, Ink O. Randomized comparative multicenter study of hydroxyethyl starch versus albumin as a plasma expander in cirrhotic patients with

tense ascites treated with paracentesis. Eur J Gastroenterol Hepatol 1998;10(1):5-10. (In eng). DOI: 10.1097/00042737-199801000-00002.

30. García-Compean D, Blanc P, Larrey D, et al. Treatment of cirrhotic tense ascites with Dextran-40 versus albumin associated with large volume paracentesis: a randomized controlled trial. Ann Hepatol 2002;1(1):29-35. (In eng).

31. Sola-Vera J, Miñana J, Ricart E, et al. Randomized trial comparing albumin and saline in the prevention of paracentesis-induced circulatory dysfunction in cirrhotic patients with ascites. Hepatology 2003;37(5):1147-53. (In eng). DOI: 10.1053/jhep.2003.50169.

32. Moreau R, Valla DC, Durand-Zaleski I, et al. Comparison of outcome in patients with cirrhosis and ascites following treatment with albumin or a synthetic colloid: a randomised controlled pilot trail. Liver Int 2006;26(1):46-54. (In eng). DOI: 10.1111/j.1478-3231.2005.01188.x.

33. Sort P, Navasa M, Arroyo V, et al. Effect of intravenous albumin on renal impairment and mortality in patients with cirrhosis and spontaneous bacterial peritonitis. N Engl J Med 1999;341(6):403-9. DOI: 10.1056/NEJM199908053410603.

34. Chen TA, Tsao YC, Chen A, et al. Effect of intravenous albumin on endotoxin removal, cytokines, and nitric oxide production in patients with cirrhosis and spontaneous bacterial peritonitis. Scand J Gastroenterol 2009;44(5):619-25. (In eng). DOI: 10.1080/00365520902719273.

35. Guevara M, Terra C, Nazar A, et al. Albumin for bacterial infections other than spontaneous bacterial peritonitis in cirrhosis. A randomized, controlled study. J Hepatol 2012;57(4):759-65. DOI: 10.1016/j.jhep.2012.06.013.

36. Thevenot T, Bureau C, Oberti F, et al. Effect of albumin in cirrhotic patients with infection other than spontaneous bacterial peritonitis. A randomized trial. J Hepatol 2015;62(4):822-30. DOI: 10.1016/j.jhep.2014.11.017.

37. Fernandez J, Angeli P, Trebicka J, et al. Efficacy of Albumin Treatment for Patients with Cirrhosis and Infections Unrelated to Spontaneous Bacterial Peritonitis. Clin Gastroenterol Hepatol 2020;18(4):963-973 e14. DOI: 10.1016/j.cgh.2019.07.055.

38. China L, Freemantle N, Forrest E, et al. A Randomized Trial of Albumin Infusions in Hospitalized Patients with Cirrhosis. N Engl J Med 2021;384(9):808-817. (In eng). DOI: 10.1056/NEJMoa2022166.

39. Philips CA, Maiwall R, Sharma MK, et al. Comparison of 5% human albumin and normal saline for fluid resuscitation in sepsis induced hypotension among patients with cirrhosis (FRISC study): a randomized controlled trial. Hepatol Int 2021;15(4):983-994. (In eng). DOI: 10.1007/s12072-021-10164-z.

40. Maiwall R, Kumar A, Pasupuleti SSR, et al. A randomized-controlled trial comparing 20% albumin to plasmalyte in patients with cirrhosis and sepsis-induced hypotension [ALPS trial]. J Hepatol 2022;77(3):670-682. (In eng). DOI: 10.1016/j.jhep.2022.03.043.

41. Caraceni P, Riggio O, Angeli P, et al. Long-term albumin administration in decompensated cirrhosis (ANSWER): an open-label randomised trial. Lancet 2018;391(10138):2417-2429. (In eng). DOI: 10.1016/s0140-6736(18)30840-7.

42. Solà E, Solé C, Simón-Talero M, et al. Midodrine and albumin for prevention of complications in patients with cirrhosis awaiting liver transplantation. A randomized placebo-controlled trial. J Hepatol 2018;69(6):1250-1259. (In eng). DOI: 10.1016/j.jhep.2018.08.006.
